# Mathematical model of hemodynamic mechanisms and consequences of glomerular hypertension in diabetic mice

**DOI:** 10.1038/s41540-018-0077-9

**Published:** 2018-12-10

**Authors:** Hari Shankar Mahato, Christine Ahlstrom, Rasmus Jansson-Löfmark, Ulrika Johansson, Gabriel Helmlinger, K. Melissa Hallow

**Affiliations:** 1 School of Chemical, Materials, and Biomedical Engineering, University of Georgia, Athens, GA USA; 2 Drug Metabolism and Pharmacokinetics, Cardiovascular, Renal, and Metabolism, Innovative Medicines and Early Development Biotech Unit, AstraZeneca, Gothenburg, Sweden; 3 Bioscience Chronic Kidney Disease, Cardiovascular, Renal, and Metabolism Innovative Medicines and Early Development Biotech Unit, AstraZeneca, Gothenburg, Sweden; 4 Quantitative Clinical Pharmacology, Innovative Medicines and Early Development Biotech Unit, AstraZeneca, Waltham, MA USA

## Abstract

Many preclinically promising therapies for diabetic kidney disease fail to provide efficacy in humans, reflecting limited quantitative translational understanding between rodent models and human disease. To quantitatively bridge interspecies differences, we adapted a mathematical model of renal function from human to mice, and incorporated adaptive and pathological mechanisms of diabetes and nephrectomy to describe experimentally observed changes in glomerular filtration rate *(GFR)* and proteinuria in db/db and db/db UNX (uninephrectomy) mouse models. Changing a small number of parameters, the model reproduced interspecies differences in renal function. Accounting for glucose and Na^+^ reabsorption through sodium glucose cotransporter 2 (SGLT2), increasing blood glucose and Na^+^ intake from normal to db/db levels mathematically reproduced glomerular hyperfiltration observed experimentally in db/db mice. This resulted from increased proximal tubule sodium reabsorption, which elevated glomerular capillary hydrostatic pressure (*P*_gc_) in order to restore sodium balance through increased GFR. Incorporating adaptive and injurious effects of elevated *P*_gc_, we showed that preglomerular arteriole hypertrophy allowed more direct transmission of pressure to the glomerulus with a smaller mean arterial pressure rise; Glomerular hypertrophy allowed a higher GFR for a given *P*_gc_; and *P*_gc_-driven glomerulosclerosis and nephron loss reduced GFR over time, while further increasing *P*_gc_ and causing moderate proteinuria, in agreement with experimental data. UNX imposed on diabetes increased *P*_gc_ further, causing faster GFR decline and extensive proteinuria, also in agreement with experimental data. The model provides a mechanistic explanation for hyperfiltration and proteinuria progression that will facilitate translation of efficacy for novel therapies from mouse models to human.

## Mathematical model of mouse diabetic kidney injury

Many drugs for diabetic kidney disease appear to work in rodents, but fail in humans, reflecting incomplete understanding of disease processes. A team led by Melissa Hallow at the University of Georgia has developed a mathematical model that explains how elevated blood glucose in diabetes causes kidney injury in mice. They first showed that normal human, rat, or mouse kidney physiology could be reproduced with the same model by changing a small number of parameters. They then showed that diabetes-induced increases in sodium reabsorption cause unintuitive changes in kidney function that increase pressure on glomerular capillaries, causing protein leakage and nephron loss. The model reproduced faster disease progression observed in diabetic mice who have had one kidney removed. This mathematical understanding of diabetic kidney injury may improve translation of novel therapies from mice to human.

## Introduction

Diabetic kidney disease is a growing health problem worldwide, yet the development of novel therapeutic treatments remain challenging. There have been multiple experimental therapies with promising efficacy attributes in preclinical studies, which have not yielded efficacy in humans.^1^ Part of the challenge is that we do not fully understand quantitative and kinetic aspects of how kidney disease in rodent models of diabetic nephropathy develops, progresses, and differs from humans. Such gaps in our understanding can result in a flawed interpretation of preclinical efficacy data of novel experimental compounds. Mathematical modeling can be a useful tool in quantitatively bridging differences in disease processes, time courses of progression, and responses to therapies across species.^2^

In humans, early diabetic kidney disease is characterized by glomerular hyperfiltration, hypertrophy of the glomerulus and tubules, and development of microalbuminuria. This early injury is followed by progressive glomerulosclerosis, overt proteinuria, nephron loss, and ultimately renal failure.^3,4^ Advanced diabetic nephropathy developing over decades in humans is difficult to address in animal models. Only a few of the available diabetic nephropathy rodent models exhibit most of the characteristics of human diabetic nephropathy, secondary to Type 2 diabetes mellitus. Mouse models with deficient leptin production (black and tan, brachyuric (BTBR) ob/ob mouse) or dysfunctional leptin signaling (db/db mouse) have shown promising results in mimicking human disease progression.^5,6^ The BTBR ob/ob mouse model develops glomerular lesions with progressive proteinuria at a young age; however, a major concern with these mice is their infertility, resulting in difficulties in breeding.^6^ The db/db mouse model demonstrates characteristics of early human diabetic kidney disease, such as hypertrophy, hyperfiltration, and albuminuria,^7^ and appears to most closely mimic the progression of mesangial matrix expansion seen in humans.^7^ However, disease progression in the db/db model, including mesangial matrix expansion, is slower than in the BTBR ob/ob and only mild proteinuria may develop.^7,8^ Uninephrectomy (UNX) at 6–8 weeks of age in db/db mice has proven to accelerate the development of late stage diabetic nephropathy, with mice exhibiting mesangial matrix expansion and overt albuminuria at an age of 20–22 weeks consistent with abnormalities of advanced human diabetic kidney disease.^3^ The db/db UNX mouse model is commonly used in preclinical drug development programs, to evaluate drug targets and compounds and to inform efficacy predictions in a human diabetic nephropathy population. Even though this animal model exhibits a majority of characteristics seen in the human population, translation of efficacy and of its time course to patients remains challenging. A quantitative mathematical disease model that incorporates common processes of renal injury, as well as between-species differences, may improve our ability to interpret data within this translation process.

We previously described a mathematical model of renal function and sodium (Na^+^) and volume homeostasis.^9^ This model was developed with data from human and rat studies. We used the model to demonstrate the tubular hypothesis of diabetic hyperfiltration,^10^ whereby hyperfiltration in early diabetes is an indirect consequence of increased proximal tubule (PT) Na^+^ reabsorption. We further showed that increased PT Na^+^ reabsorption in diabetes contributes to increased glomerular hydrostatic pressure (especially when regulation of tubular Na^+^ handling is impaired) and proposed that this may represent an initiating step in diabetic kidney disease progression.^11^

In the present modeling study, we first show that the same mathematical model of normal renal physiology may be reparameterized to reproduce renal function in mice, rats, and humans. We then extend this model to represent adaptive and pathological changes that occur following development of diabetes and nephrectomy, focusing on the db/db and db/db UNX mouse models of kidney disease.

## Results

### Translating normal renal function across species

**Fig. 1:**
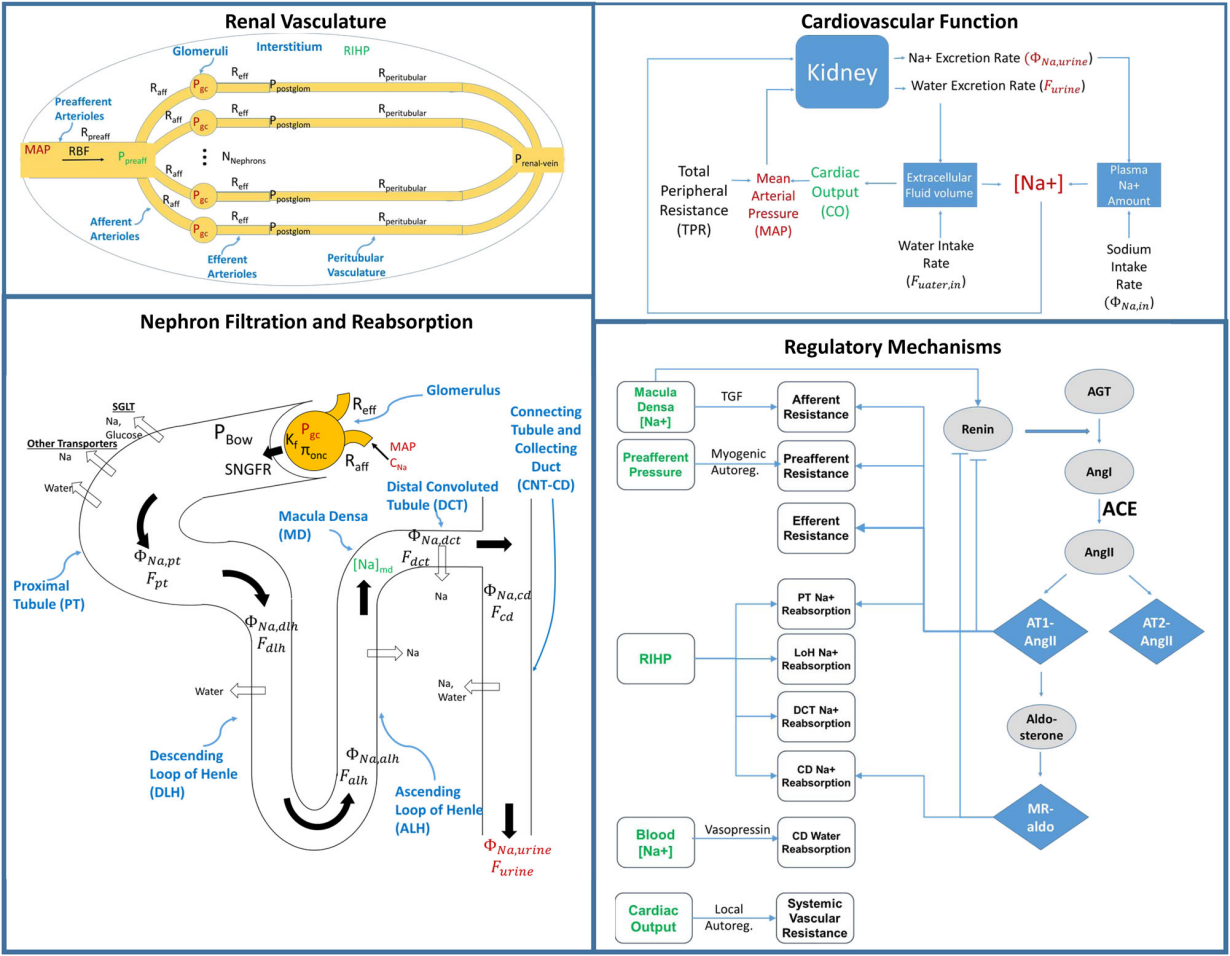
Schematic representation of the base model of renal function. (Top Left) The renal vasculature is modeled by a single preafferent resistance vessel flowing into N parallel nephrons. Bottom Left) Na^+^ and water filtration through the glomerulus are modeled according to Starling’s law. Na^+^ and water are reabsorbed at different fractional rates in the proximal tubule, loop of Henle, distal convoluted tubule, and connecting tubule/collecting duct, and Na^+^ and water excretion rates are determined from unabsorbed Na^+^ and water. Top right) Na^+^ and water excretion feed into the cardiovascular portion of the model, where the balance between excretion and intake determines extracellular fluid volume, plasma Na^+^ concentration, cardiac output (CO) and ultimately mean arterial pressure (*MAP*). Na^+^ concentration and *MAP* feed back into the renal model (left), closing the loop. Bottom Right) Multiple regulatory mechanisms, including the renin-angiotensin-aldosterone system (RAAS), tubulo-glomerular feedback (TGF), myogenic autoregulation, renal interstitial hydrostatic pressure (RIHP) regulation of tubular Na^+^ reabsorption, vasopressin regulation of tubular water reabsorption, and local blood flow autoregulation, provide feedbacks on model variables, to maintain or return homeostasis

The core model of renal function, illustrated in Fig. 1, was originally developed to describe normal human physiology.^9,11^ We first aimed to reparametrize this model to represent normal rat and mouse renal function. The key underlying processes of renal blood flow, filtration, reabsorption, excretion, and systemic volume homeostasis were assumed to be structurally the same across different species. Thus, model equations remained unchanged across species; only model parameter values were changed. These parameter values, along with measureable model variables (e.g., *glomerular filtration rate [GFR], mean arterial pressure [MAP], cardiac output [CO])* were determined, for each species, from literature sources. Resistance parameters were calculated based on other parameters and variables, as described previously.^9^ For instance, rather than specifying total peripheral resistance, it was instead calculated for each species based on that species’ normal mean arterial pressure and cardiac output.

Model parameter values for each species, given in Table 1, were used to reproduce the phenotypic behavior for each species, as listed in Table 2. Many morphologic and functional parameters are conserved across species. Arteriole and tubular diameters, the ultrafiltration coefficient, and plasma concentrations of glucose and Na^+^ are highly consistent across species. Other parameters vary widely across species, in accordance with body weight. The number of nephrons (*N*_nephrons_), the extracellular fluid volume (*ECF*), Na^+^ (*Na*_in_), water (*W*_in_), and food (*F*_in_) intakes all vary by orders of magnitude, presumably partly influenced by species size.

**Table 1:** Model parameters for human, rat, and mouse

Parameter	Symbol	Units	Human	Rat	Mouse
Parameters that differ by species					
Number of nephrons	*N*_nephrons_	–	2 *×* 10^6^	50,000	10,000
Sodium intake	*Na*_in_	mmol/day	100	2	0.576
Water intake	*Wa*_in_	L/day	2	0.025	0.005
Proximal tubule length	*L*_*pt*_	mm	14	5	2.2
Plasma protein concentration	*C*_*p*_	g/dl	7	6.2	3.4
Parameters conserved across species					
Plasma glucose level		mg/dl	90	90	90
Plasma sodium concentration	*C*_Na_	mmol/L	140	140	140
Glomerular ultrafiltration coefficient	*K*_f,0_	nl/min-mmHg	3.9	3.9	3.9
Albumin sieving coefficient	*K*_albumin_	–	0.0006	0.0006	0.0006
Renal capacity for albumin reabsorption	*RC*_albumin_	mg/min	2.5 *×* 10^-6^	2.5 *×* 10^-6^	2.5 *×* 10^-6^
Efferent arteriole diameter	*D*_eff_	µm	11	10.5	10.5
Afferent arteriole diameter	*D*_aff_	µm	14.5	14	14
Proximal tubule diameter	*D*_pt_	µm	27	27	27
Renal threshold for glucose reabsorption	RT_g_	mg/dl	180	180	180
Fractional PT Na reabsorption		*η*_pt,non-SGLT2_	0.76	0. 76	0. 76
Calculated parameters					
Renal vascular resistances	*R*_rvr_	mmHg-L/min	81	8727	58,180
Peritubular resistance	*R*_peri_	mmHg-L/min	5.766	537	2700
Preafferent resistance	*R*_prea_	mmHg-L/min	14	3500	23,000
Total peripheral resistance	*R*_rpr_	mmHg-L/min	18.6	258	1114
Parameters of kidney adaptation and injury					
Maximum afferent arteriole diameter increase	*ΔD*_a,max_				25%
Maximum increase in surface area	*ΔSA*_max_				50%
Maximum increase in permeability	*Δ*Perm_max_				100%
Time constant for increase in afferent diameter	*τ*_daa_				4 *×* 10^6^
Time constant for increase in surface area	*τ*_SA_				750
Time constant for increase in permeability	*τ*_perm_				4 *×* 10^4^
Albumin scaling factor	*A*_albumin_				4 *×* 10^4^

**Table 2:** Observed (literature) and simulated phenotypic behavior in each species

Output parameters			Human		Rat		Mouse		References
	Units	Symbols	Sim.	Lit.	Sim.	Lit.	Sim.	Lit.	^15– 23, 42– 50^
Glomerular Filtration Rate	ml/min	*GFR*	105	105 ± 15	2.5	1.9 ± 0.7	0.3	0.35 ± 0.1	
Single Nephron GFR	nl/min	*SNGFR*	55	52 ± 8	50	50 ± 10	29	33 ± 5	
Sodium Excretion	mEq/day	*Na*_*ex*_	100	100 ± 15	2	2 ± 0.6	0.576	0.58 ± 0.02	
Mean Arterial Pressure	mmHg	*MAP*	93	90 ± 20	103	98 ± 5	98	98 ± 5	
Glomerular Pressure	mmHg	*P*_*gc*_	60	60 ± 15	42	38 ± 5	37.5	38 ± 5	
Cardiac Output	l/min	*CO*	5	5 ± 0.7	0.4	0.4 ± 0.2	0.088	0.08 ± 0.04	
Renal Blood Flow	ml/Min	*RBF*	1100	10^3^ ± 300	11	10 ± 3	1.8	1.6 ± 0.25	

Pressures (e.g., MAP, glomerular hydrostatic pressure, tubular pressures, etc.) are also quite similar across species, while flow rates (*CO, RBF, GFR*) vary by orders of magnitude. Interestingly, while *GFR* varies widely across species, single nephron *GFR* is highly conserved. In larger species, a higher *GFR* is achieved through an increased number of nephrons, while single-nephron flow and pressure metrics are remarkably similar.

### Composite profile of disease progression in db/db and db/db UNX mice

**Fig. 2:**
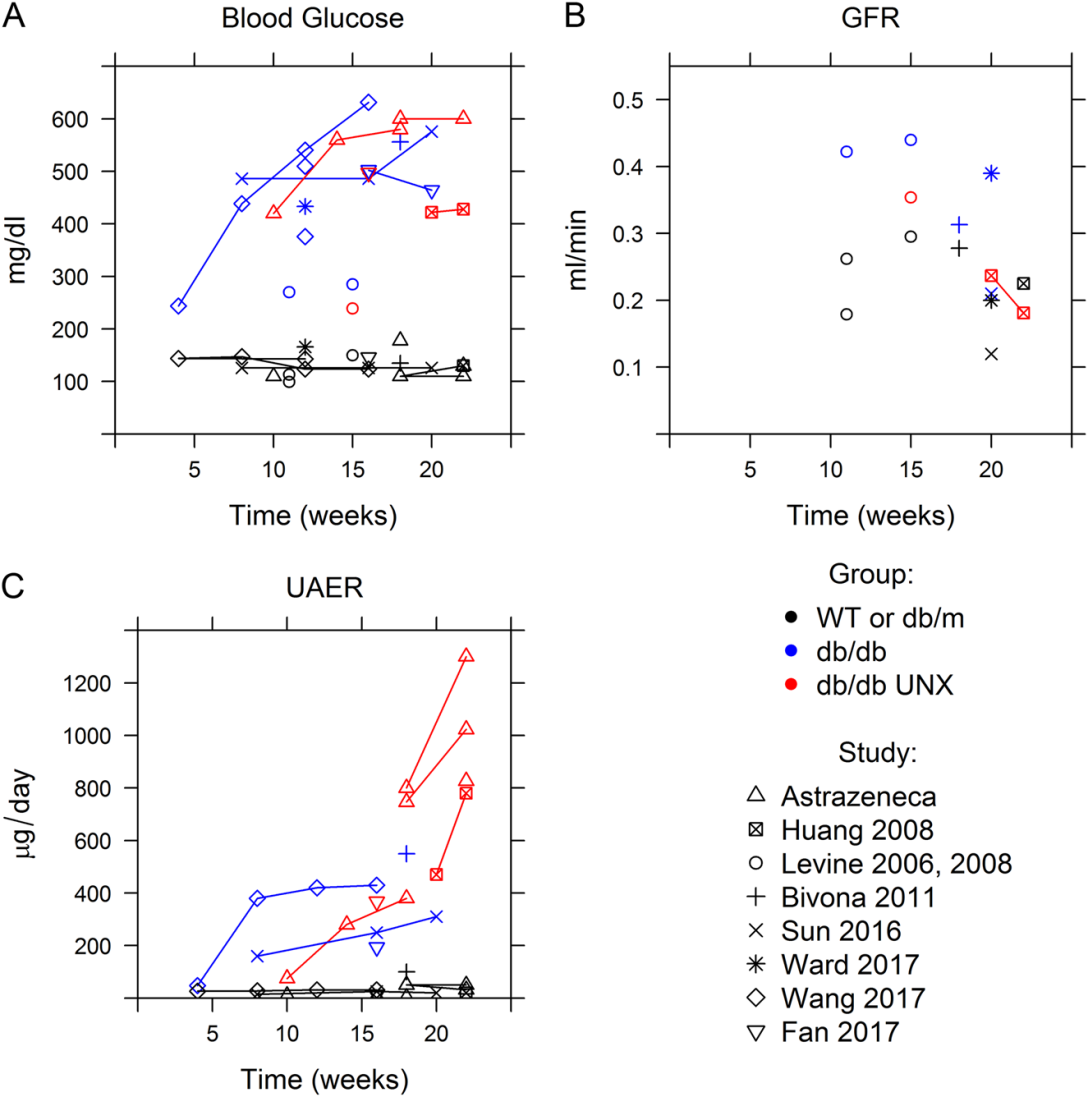
Blood glucose, *GFR*, and UAER over time in db/db mice with or without uninephrectomy (UNX–performed between 8–10 weeks of age), compared to WT or db/m mice. Data were obtained from published studies (Huang 2008,^12^ Levine 2006,^46^ Levine 2008,^13^ Bivona 2011,^21^ Sun 2016,^47^ Ward 2017,^48^ Wang 2017,^49^ and Fan 2017^50^) and unpublished AstraZeneca reports of additional studies conducted according to protocols of Huang 2008^12^

We next sought to use the model to mechanistically and quantitatively describe the pathophysiological processes that produce disease progression observed in obese leptin receptor deficient db/db mice. There is considerable variability in the levels of blood glucose, proteinuria, and *GFR* reported among studies in db/db mice. Thus, rather than relying on a single study to inform the model, a composite dataset was developed from all identified published studies for which either urinary albumin excretion rate, *GFR*, or both were reported in db/db mice, with or without UNX. In addition, data from three unpublished AstraZeneca studies, conducted according to the protocols of Huang 2008,^12^ were included. These data are summarized in Fig. 2. In all studies in which UNX mice were included, uninephrectomy was conducted between weeks 8 and 10. Blood glucose was stable in WT and db/m mice, but increased over time in db/db mice (both UNX and non-UNX) to reach levels between 400 and 600 mg/dl in most studies. *GFR* was elevated in db/db mice compared to wild type and db/m, as expected. Although few studies in db/db UNX mice reported *GFR*, one study^13^ found *GFR* higher than WT and db/m but lower than db/db non-UNX at week 15, and another study^12^ found *GFR* similar to WT and falling from week 20–22. These data suggest that *GFR* recovers after UNX to near levels of non-UNX, but then declines more rapidly over time compared to db/db non-UNX mice. The urinary albumin excretion rate (UAER) increased over time in db/db mice, and increased more rapidly in db/db UNX mice following uninephrectomy.

### Modeling hyperfiltration in the db/db mouse model

To mathematically reproduce functional renal changes observed in db/db non-UNX mice, blood glucose was allowed to increase over time, in the model, up to 500 mg/dl, consistent with data shown in Fig. 2. Sodium and water were increased by 1.5 and 5.0-fold, respectively, consistent with reported levels from studies described above. Data from these studies also point to minimal urinary glucose excretion (UGE) in animals with plasma glucose concentrations of less than 400 mg/dl (note: this is a much higher capacity than is typically observed in humans, where glucose excretion occurs for plasma levels above ~250 mg/dl).^14,15^ This suggests that, as animals become diabetic, the PT capacity for glucose reabsorption increases sufficiently, so that all filtered glucose is still reabsorbed, at least up to 400 mg/dl. Thus, the renal threshold for glucose reabsorption, RTg, was increased to 400 mg/dl. Simulations were first conducted without considering any additional adaptive or maladaptive changes.

**Fig. 3:**
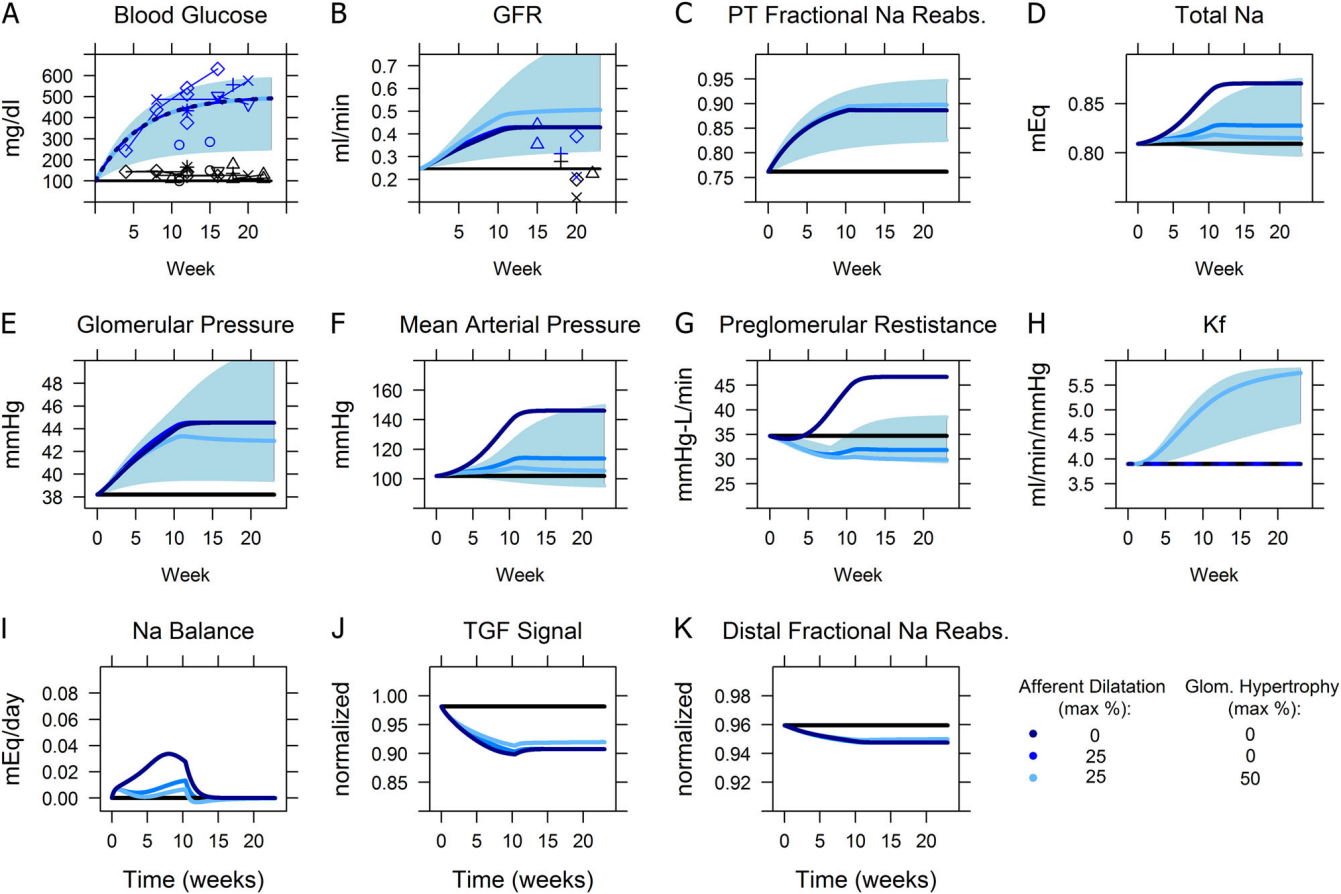
Simulated responses to development of diabetes (simulated as a progressive increase in blood glucose, sodium intake, and renal threshold for glucose excretion) in db/db mice, and the effect of afferent dilatation and glomerular hypertrophy. Solid lines: simulations (light, medium and dark blue solid lines: db/db mice, black line: simulated non-db mice) points: experimental data (blue: db/db mice, black: WT or db/m mice). See Fig. 2 for data references. Shaded area: range of response as blood glucose varied from 250 to 600 mg/dl, under conditions of 25% afferent dilatation and 50% glomerular hypertrophy. Increasing blood glucose **a** and the associated increase in PT sodium reabsorption **c** caused sodium retention **d** and increases in mean arterial pressure **f** and glomerular pressure **g**, until *GFR* increased sufficiently to return sodium balance **i** and prevent further sodium accumulation. Afferent dilatation prevented preglomerular resistance **g** from rising and allowed the necessary hyperfiltration to be achieved at lower MAP, while glomerular hypertrophy **h** allowed it to be achieved at lower glomerular pressure

As shown in Fig. 3 (dark blue color), as glucose increased over time, *GFR* also increased, although the increase slowed down once glucose reached the RTg threshold value of 400 mg/dl. The reason for this phenomenon is as follows: increased plasma glucose results in more glucose being filtered through the glomerulus. For glucose levels below the threshold RTg value of 400 mg/dl,^14,15^ all the excess glucose is reabsorbed in the PT. Since Na^+^ and glucose reabsorption processes are coupled through the SGLT2transporter, PT fractional Na^+^ reabsorption is increased as well (Fig. 3c), producing a sodium imbalance. The only ways to restore Na^+^ balance are to: a) reabsorb less Na^+^; or b) filter more Na^+^. While the model accounts for some compensation through changes in tubular Na^+^ reabsorption, these adaptations are not sufficient.^11^ Thus, over time, Na^+^ and water were retained (Fig. 3d), driving up mean arterial pressure and glomerular pressure (Fig. 3e, f), thus increasing *GFR* up until sodium balance is returned. We previously described this mechanism for diabetic hyperfiltration in detail.^11^

### Kidney adaptation and injury in response to diabetes and glomerular hypertension

**Fig. 4:**
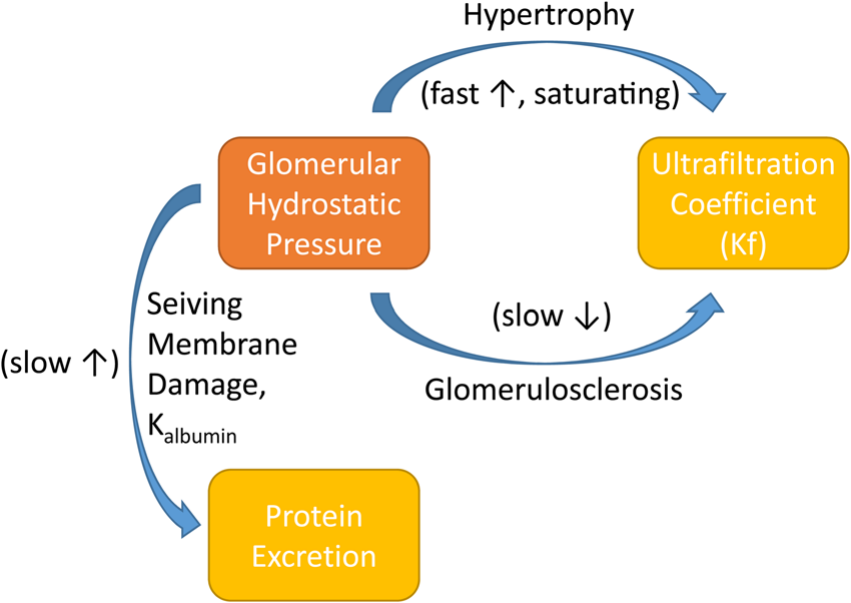
Modeling of adaptive and maladaptive (pathological) effects of elevated glomerular hydrostatic pressure. When glomerular pressure is increased above normal, glomeruli adapt by growing, thereby increasing the filtering surface area and the associated ultrafiltration coefficient *K*_*f*_ in a relatively fast (over the course of weeks) saturating process. At the same time, elevated glomerular pressure initiates glomerulosclerosis, a much slower and non-saturating process that increases the basement membrane thickness, thus reducing permeability and lowering *K*_*f*_. Elevated glomerular pressure also damages the podocytes, effectively increasing the protein sieving coefficient

The simulations described above showed that diabetes can produce an increase in *GFR* and glomerular hypertension, but did not yet account for adaptive and maladaptive changes accompanying these effects. These simulations predicted a large increase in MAP (40 mmHg), as well as an associated increase in preglomerular vascular resistance, as myogenic autoregulation attempts to prevent transmission of increased systemic pressure to the glomerulus—but db/db mice do not consistently develop hypertension. Afferent arteriole diameters have been found to be 25% larger in db/db mice compared to db/m.^16^ The same study found that myogenic autoregulation in db/db mice was intact, although studies in other models of diabetes have shown autoregulatory impairment.^17^ Increased afferent diameter may allow MAP to be more directly transmitted to the glomerulus. In addition, in both humans and animal models of diabetes, the early diabetic kidney is characterized by glomerular and tubular hypertrophy.^18,19^ Similar patterns of hypertrophy are observed in the remaining kidney following nephrectomy in both mice and humans.^13,20^ This hypertrophy response is likely an adaptation to accommodate increased demands for filtration and reabsorption. Across species, this process occurs quickly (over weeks to months), and glomerular volume increases as much as 50%.^13,21,22^ As diabetic kidney disease progresses, glomerulosclerosis, the scarring or thickening of the glomerular basement membrane, occurs, and podocytes become damaged, all leading to proteinuria. Excess protein in the tubules accelerates the rate of disease progression, likely by stimulating inflammation, resulting in tubular injury, fibrosis, and ultimately nephron loss.^23^ Although multiple factors may be involved, elevated glomerular hydrostatic pressure is believed to be a major driver of these processes.^24^ We have incorporated these adaptive and pathological consequences of elevated glomerular hydrostatic pressure into the model, through changes over time in model parameters, as illustrated in Fig. 4.

### Afferent Arteriole dilatation

When afferent arterioles were allowed to increase in diameter up to 25% in response to elevated blood glucose, the model showed that the same rise in *GFR* was achieved with a much smaller increase in MAP compared to the case without afferent dilatation (Fig. 3, medium blue line). Because preglomerular resistance did not rise, increases in MAP were more directly transmitted to the glomerulus, allowing *GFR* to increase and sodium balance to be restored at a much lower systemic pressure.

### Glomerular hypertrophy

The glomerular ultrafiltration coefficient K_f_ represents both the permeability and the surface area of the glomerular membrane. When K_f_ was allowed to increase over time in response to changes in glomerular pressure (up to limit of 50%, the maximum increase observed in diabetic and/or nephrectomized rats, mice, and humans^13,21,22^), to represent increased glomerular filtration surface area with hypertrophy, glomerular pressure increased less while *GFR* increased further, suggesting that hypertrophy is an adaptive response that limits diabetes-induced increases in glomerular pressure (Fig. 3–light blue). The time constant τ_SA_, which determines the speed at which glomerular hypertrophy occurs, was set such that glomerular hypertrophy occurred quickly and would reach equilibrium within 3 months.

There is a large degree of variability in glucose levels among studies in db/db mice. The shaded regions in Fig. 3 show the predicted ranges for each variable as steady-state glucose varied from a lower bound of 250 to an upper bound of 600 mg/dl.

**Fig. 5:**
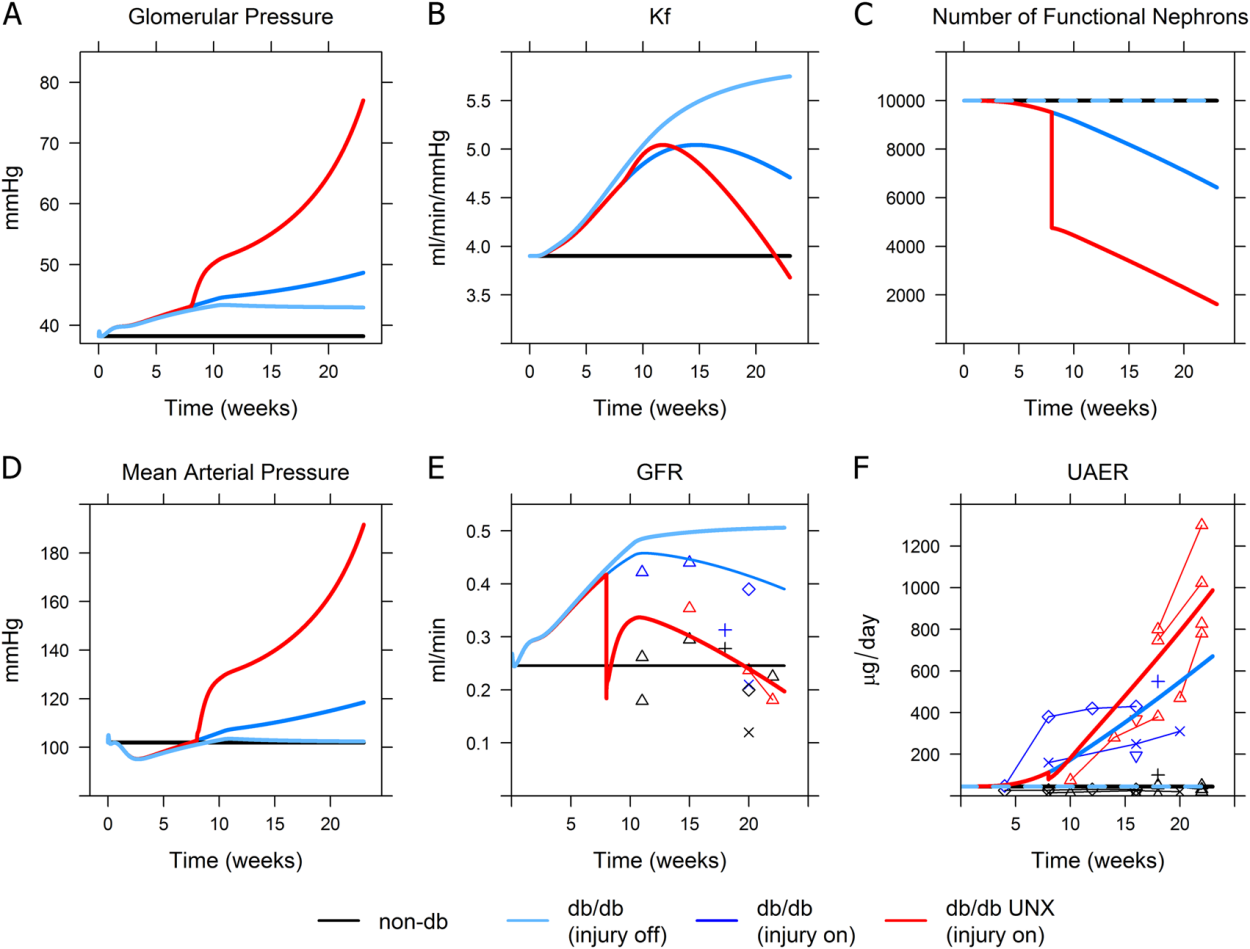
Simulated effects of pressure-induced nephron injury and uninephrectomy on disease progression in db/db mice. Solid lines: simulations (black: non-db, light blue – db/db non-UNX with injury effects turned off, dark blue–db/db non-UNX with injury effects turned on, red–db/db UNX with injury effects turned on). Points: experimental data (black non-db, blue – db/db, red – db/db UNX). See Fig. 2 for data references. In db/db non-UNX mice, when injury effects were turned on, elevated glomerular pressure (**a**) caused K_f_ to peak and then begin to decrease (**b**), as the effect of glomerular hypertrophy saturated while the effect of glomerosclerosis grew; elevated glomerular pressure also drove a decrease in nephron number (**c**) and an increase in glomerular albumin sieving. *GFR* (**e**) increased over time but eventually began to decrease slowly around week 10, while glomerular pressure (**a**) and UAER (**f**) progressively increased, in agreement with experimental data. When UNX was simulated at 8 weeks, *GFR* initially dropped but recovered before beginning to progressively decline. This progression was driven by a further increase in glomerular pressure, and an accelerated rate of glomerulosclerosis (K_f_ decrease) and nephron loss

Figure 5 (blue lines) shows the effects of allowing glomerular pressure to drive a decrease in K_f_ due to glomerulosclerosis, a decrease in the number of nephrons, and an increase in the glomerular protein sieving rate over time. Parameters governing these relationships, as given in Table 1, were chosen in order to best fit the experimental data. Figures S1–S3 in the Supplemental Material show the sensitivity of the simulation results to varying values for these time constants.

K_f_ increased during the first few weeks due to hypertrophy, but eventually began to decrease as hypertrophy reached its maximum and effects of glomerular pressure on glomerulosclerosis accumulated. The number of nephrons also began to decline over time, as glomerular pressure began to increase. As *K*_f_ and nephron number decreased, *GFR* began to decrease, although it remained above normal levels. While *GFR* decreased, the glomerular pressure required to achieve this *GFR* increased further.

### Modeling hyperfiltration and proteinuria in the db/db UNX mouse model

Progression of kidney disease and development of overt proteinuria in db/db mice are accelerated by uninephrectomy. We next tested whether our mathematical model could reproduce and explain changes in *GFR* and proteinuria that occur in db/db UNX mice, using parameters for disease progression determined above for db/db mice. To simulate db/db with UNX, we simulated diabetes as before, and then reduced the number of nephrons by half after 8 weeks of simulation time (UNX in the studies included in Fig. 2 were all conducted between weeks 8 and 10).

As shown in Fig. 5, following uninephrectomy at week 8, *GFR* was acutely reduced, but increased towards prior levels (and above non-diabetic levels) over the next few weeks, consistent with experimental observations. Because the increase in *K*_*f*_ due to hypertrophic surface area increase was already near its upper limit following induction of diabetes (glomerular volume increases by at most 50% in diabetes^13,21^), *GFR* could not increase further. At the same time, elevated glomerular pressure began driving glomerulosclerosis—a slower process of matrix deposition that thickens the glomerular basement membrane and reduces glomerular permeability. In addition, elevated glomerular pressure drove additional nephron loss over time. As *K*_f_ and nephron number decreased, glomerular pressure increased even further to maintain the *GFR* necessary for Na^+^ balance. The increase in glomerular pressure increased protein sieving and UAER. The magnitude and time course of UAER changes were consistent with the experimentally observed, higher level of and progressively increasing proteinuria in uninephrectomized mice.

## Discussion

In this study, we adapted a mathematical model of renal physiology and function from human to mice, and incorporated adaptive and pathological mechanisms of diabetes and nephrectomy to describe changes in *GFR* and proteinuria observed in experimental db/db and db/db UNX mouse models. By changing a small number of parameters describing renal morphology, water and Na^+^ intake, as well as plasma protein concentration, the model reproduced differences and similarities in normal renal function between species. Nephron structure is conserved across species, in terms of morphology, as well as arteriole and tubule diameters)^25^; significant cross-species differences are numbers of nephrons, which vary by orders of magnitude, and smaller differences in tubule segment lengths. Thus, while total *GFR*, RBF, and RVR may vary by order(s) of magnitude between species, at the single-nephron level, pressures and flow rates (and associated mechanical stretch and shear stress) experienced by cells within the glomeruli and tubules, as well as single-nephron filtered loads of reabsorbed substances such as Na^+^ and glucose, appear to be similar across mouse, rat, and human mammalian species. This suggests there is an optimal mechanical and biochemical environment for glomerular and tubular cells and tissues. Indeed, nephron morphogenesis has been shown to be directed in part by fluid flow.^26^ Thus, it is to be expected that alterations which impact pressure and flow rate would lead to adaptations. Glomerular volume has been found to vary inversely with nephron number, which suggests hypertrophy is a compensatory response to allow for *GFR* to be maintained.^27,28^ Based on this, we considered, in this analysis, the role of glomerular hypertrophy as a response to elevated glomerular capillary pressure.

We previously demonstrated, mathematically [12], the tubular hypothesis of diabetic hyperfiltration:^10,29^ increased filtration is a necessary consequence of increased PT Na^+^ reabsorption in diabetes, and is required to restore Na^+^ balance. It is achieved through a combination of TGF, changes in Bowman’s pressure, and Na^+^ retention, leading to increased net filtration pressure and glomerular hypertension. A key physiological principle incorporated in the model is the requirement for Na^+^ balance. When PT Na^+^ reabsorption is increased, a Na^+^ imbalance is generated, which is compensated for by a combination of increased filtration and decreased reabsorption through other mechanisms [12]. In this paper, we extended the model to directly account for reabsorption of filtered glucose and Na^+^ through SGLT2, and we showed that by changing only blood glucose and Na^+^ intake from normal to db/db levels, the model reproduced the glomerular hyperfiltration observed in db/db mice. Hyperfiltration was initially achieved through elevations in mean arterial pressure and glomerular capillary hydrostatic pressure. Simulations demonstrated that increases in preglomerular arteriole diameters observed in diabetes prevent an excessive rise in mean arterial pressure, yet allow systemic blood pressure to be transmitted more directly to the glomerulus. Glomerular hypertrophy (represented as an increase in *K*_f_ due to increased glomerular surface area) in response to elevated glomerular pressure served to mitigate glomerular pressure increases, while maintaining the same *GFR*.

Similarly, uninephrectomy, represented in the model by reducing the number of nephron by half, initially produced a Na^+^ imbalance, which was quickly restored by both hyperfiltration of remaining nephrons (increased *SNGFR*, driven by a further increase in glomerular pressure) and decreased distal reabsorption, so that *GFR* returned to near normal. Elevated glomerular pressure is assumed to drive hypertrophy, but hypertrophic growth is not unlimited. Following diabetes initiation, simulations suggest that hypertrophic growth is already near maximum. After nephrectomy, as Na^+^ is retained and glomerular pressure begins to increase, *K*_f_ cannot increase further to compensate. Thus, glomerular pressure rises and remains elevated. Elevated glomerular pressure then rapidly begins to damage podocytes, allowing protein filtration and producing overt proteinuria, as is observed in db/db UNX mice. The model also predicts small rises in mean arterial pressure in the db/db mice, and much larger increases in db/db UNX mice. Experimentally, blood pressure is not often measured in these animals, but studies which did measure it report highly variable levels of increases, with changes in blood pressure ranging from negligible to as much as 40 mmHg.^30,31^

Our model-based simulations illustrate a general concept that is not specific to diabetic hyperfiltration: to maintain homeostasis, there is a level of *GFR* that needs to be achieved to maintain Na^+^ excretion sufficient for Na^+^ balance. *GFR* is proportional to *K*_f_ and net filtration pressure. Thus, as *K*_f_ increases, the necessary *GFR* (and thus Na^+^ balance) can be achieved with a lower glomerular hydrostatic pressure. This supports a role for glomerular hypertrophy as an adaptive response, to prevent exposure of glomeruli to deleterious elevations in glomerular pressure.

Glomerular hypertension is associated with progressive deposition of excess glomerular extracellular matrix and formation of glomerular lesions, and therapies such as ACE inhibitors, which reduce glomerular hydrostatic pressure, have been shown to slow this process.^32,33^ In db/db mice, this occurs rather slowly, with substantial glomerular basement membrane (GBM) thickening occuring over 6–12 months.^7^ In db/db UNX mice, it occurs much faster, with significant GBM thickening by 22 weeks. In our model, glomerular hypertension causes slow but cumulative and irreversible reductions in membrane permeability over time. We recognize that other factors, including inflammation and release of growth factors by mesangial cells and podocytes, may contribute as well. However these factors were not considered in the current model. In the simulated db/db mice, the hypertrophic response dominated, and the net increase in *K*_f_ allowed glomerular pressure to be normalized, with very little loss of membrane permeability. However, in the simulated db/db UNX model, once the hypertrophic increase in surface area reached its limit, *K*_f_ began to decrease. As *K*_f_ decreased, glomerular hydrostatic pressure had to increase further in order to maintain *GFR* and Na^+^ balance. The further increase in glomerular pressure led to more proteinuria and further GBM thickening. Thus, the model provides an elegant mechanistic explanation for hyperfiltration, progression of proteinuria and GBM thickening observed in db/db UNX mice.

This analysis focuses on hemodynamic mechanisms of kidney injury in diabetes. Diabetes being an overly complex disease process, many other factors, including inflammatory, neurohumoral, and genetic ones are likely important.^34^ The model also used a simplified representation of renal structure and function; additional complexities such as normal variability in nephron number or differences in architectural organization across species were not considered. Also, we did not attempt to model the contribution of various transporters in detail. This has been done quite elegantly by others,^35–37^ and we believe that the current representation was sufficient to explore the questions addressed in this study.

In conclusion, we adapted a mathematical model of renal function from human to mice, and incorporated adaptive and pathological mechanisms of diabetes and nephrectomy to describe changes in *GFR* and proteinuria observed in the experimental db/db and db/db uninephrectomy (UNX) mice. The model was able to reproduce experimentally observed trends, and thus provides a mechanistic explanation for the hyperfiltration and proteinuria responses observed in db/db and db/db UNX mice. Such a quantitative, mechanistically-oriented disease model may well serve as a platform for quantitatively exploring pharmacological mechanisms in a preclinical setting and for evaluating translational aspects of experimental drug effects from the db/db UNX model to specific diabetes and renal disease patient populations.

## Methods

The base model of renal function is summarized in Fig. 3, and described in detail in ref. ^38^ Full model equations are included in the Supplemental Material. Briefly, this model describes key physiological processes involved in renal function and their roles in maintaining Na^+^ and water homeostasis. It describes blood flow, resistance, and pressures through the renal vasculature; filtration of water and Na^+^ through the glomerulus; Na^+^ and water reabsorption, flow rates, and pressures in each tubular segment; Na^+^ and water excretion; Na^+^ and water balance and its effects on interstitial fluid volume and blood pressure. Key regulatory feedback mechanisms are incorporated in the model, including the renin-angiotensin-aldosterone system (RAAS), TGF, myogenic autoregulation, effects of renal interstitial hydrostatic pressure (RIHP) on regulation of tubular Na^+^ reabsorption, vasopressin regulation of tubular water reabsorption, and local blood flow autoregulation.

We here extended this model to: (1) account for glucose filtration, reabsorption through SGLT2, and excretion, (2) explicitly account for the coupling of Na^+^ with glucose reabsorption through SGLT2, (3) account for filtration, reabsorption, and excretion of albumin, and (4) simulate adaptive and maladaptive changes in the glomerulus, in response to increased glomerular pressure (afferent and glomerular hypertrophy, glomerulosclerosis, podocyte damage).

In this model (see Fig. 1), single nephron glomerular filtration rate (SNGFR) is defined according to Starling’s equation, where *K*_f_ is the glomerular ultrafiltration coefficient, *P*_gc_ is glomerular capillary hydrostatic pressure, *P*_Bow_ is pressure in the Bowman’s space, and *π*_go-avg_ is average glomerular capillary oncotic pressure:

1 $$SNGFR = K_{\mathrm{f}}(P_{\mathrm{{gc}}} - P_{{\mathrm{Bow}}} - {\it{\pi }}_{{\it{{\mathrm{go - avg}}}}})$$

The total *GFR* is then the *SNGFR* multiplied by the number of nephrons:

2 $$GFR = SNGFR \ast N_{\mathrm{{nephrons}}}$$

### Glucose filtration and reabsorption

Glucose is filtered freely through the glomerulus, so that single nephron filtered glucose load is:

3 $${\it{\phi }}_{\mathrm{{glu,filtered}}} = SNGFR \ast C_{\mathrm{{glu}}}$$

where *C*_glu_ is the plasma glucose concentration.

Glucose reabsorption occurs exclusively in the PT through Na^+^ glucose cotransporters (SGLT). SGLT2 in the S1 and S2 segments of the PT reabsorbs 90 to 97% of filtered glucose, while SGLT1 in the S3 segment reabsorbs the remaining 3 to 10%.^10,39^ As long as the filtered glucose load remains below the reabsorptive capacity of SGLT in the PT, glucose is nearly completely reabsorbed, and excreted glucose is negligible. However, at high plasma concentrations, filtered glucose can exceed the kidney’s capacity for reabsorption, and the excess glucose is excreted. The plasma concentration at which filtered glucose exceeds the renal capacity for glucose reabsorption has been defined as the renal threshold for glucose excretion, *RT*_G_ . The renal capacity for glucose reabsorption, or *RC*_glucose_, is given by:

4 $$RC_{\mathrm{{glucose}}} = RT_G \ast SNGFR$$

The rate of glucose reabsorption is then:

5 $${\it{\phi }}_{\mathrm{{glu,reabs}}} = {\mathrm{min}}({\it{\phi }}_{\mathrm{{glu,filtered}}},RC_{\mathrm{{glucose}}})$$

Any non-reabsorbed glucose is then excreted, so that the rate of urinary glucose excretion (*R*_UGE_) is:

6 $$R_{\mathrm{{UGE}}} = {\it{\phi }}_{\mathrm{{glu,filtered}}} - {\it{\phi }}_{\mathrm{{glu,reabs}}}$$

### Na^+^ filtration and reabsorption in the PT

Similarly to glucose, Na^+^ is freely filtered across the glomerulus, so that the single nephron-filtered Na^+^ load is given by:

7 $${\it{\phi }}_{\mathrm{{Na,filtered}}} = SNGFR \ast C_{\mathrm{{Na}}}$$

where *C*_Na_ is the plasma Na^+^ concentration.

Assuming that glucose reabsorption through SGLT1 is small compared to reabsorption through SGLT2, the rate of Na^+^ reabsorption through SGLT2 is approximately equal to the rate of glucose reabsorption:

8 $$\phi _{\mathrm{{Na,reabs}} - SGLT2} = \phi _{\mathrm{{glu,reabs}}}$$

Total PT Na^+^ reabsorption is then given by:

9 $${\it{\phi }}_{\mathrm{{Na,reabs}} - PT} = {\it{\phi }}_{\mathrm{{Na,filtered}}} \ast \eta _{\mathrm{{pt,non}} - SGLT2} + {\it{\phi }}_{\mathrm{{Na,reabs}} - SGLT2}$$

where *η*_pt,non-SGLT2_ is the fractional rate of PT reabsorption through mechanisms other than SGLT2. Na^+^ flow rate out of the PT is then:

10 $${\it{\phi }}_{\mathrm{{Na,out}} - PT} = {\it{\phi }}_{\mathrm{{Na,filtered}}} - {\it{\phi }}_{\mathrm{{Na,reabs}} - PT}$$

Na^+^ reabsorption along the rest of the tubule is modeled as in ref. ^11^

### Albumin filtering and excretion

The rate of albumin filtration is a function of SNGFR, the plasma albumin concentration *C*_albumin_, and the sieving coefficient *K*_albumin_, as described in^40^:

11 $${\it{\phi }}_{\mathrm{{albumin,filtered}}} = K_{\mathrm{{albumin}}} \ast SNGFR \ast C_{\mathrm{{albumin}}}$$

The proximal tubule has the capacity to reabsorb a limited amount of filtered albumin (*RC*_albumin_), beyond which excess albumin is excreted.

12 $${\it{\phi }}_{\mathrm{{albumin,reabs}}} = \min \left( {{\it{\phi }}_{\mathrm{{albumin,filtered}}} \ast \eta _{\mathrm{{albumin}}},RC_{\mathrm{{albumin}}}} \right)$$

The urinary albumin excretion rate (UAER) is then:

13 $$UAER = \left( {{\it{\phi }}_{\mathrm{{albumin,filtered}}} - {\it{\phi }}_{\mathrm{{albumin,reabs}}}} \right) \ast N_{\mathrm{{nephrons}}}$$

### Kidney adaptation and injury in response to diabetes and glomerular hypertension

Afferent arteriole diameter is increased in db/db mice, although the precise underlying mechanism has not been established. Thus, we modeled the dilatation as a function of blood glucose, without prescribing intermediate mechanisms. The arteriole diameter *D*_*aa*_ was allowed to increase up a maximum *∆D*_aa_ of 25%, and the rate of increase was proportional to the difference between blood glucose (BG) and nominal blood glucose of 90 mg/dl, with a time constant *τ*_daa_.

19 $$\frac{d}{{dt}}\left( {\Delta D_{\mathrm{{aa}}}} \right) = \left( {\Delta D_{\mathrm{{aamax}}} - \Delta D_{\mathrm{{aa}}}} \right) \ast \frac{{{\mathrm{max}}(BG - BG_{\mathrm{{nom}}},0)}}{{\tau _{\mathrm{{daa}}}}}$$

We assumed that, when glomerular capillary hydrostatic pressure *P*_gc_ rises above some normal limit *P*_gc,0_, it begins to drive both adaptive and pathophysiological changes in the glomerulus. The magnitude of this damage signal is defined as:

14 $${\mathrm{GP}}\_{\mathrm{damage}}\_{\mathrm{effect}} = {\mathrm{max}}\left( {\frac{{P_{\mathrm{{gc}}}}}{{P_{\mathrm{{gc}},0}}} - 1,0} \right)$$

The ultra-filtration coefficient *K*_*f*_, in Eq. 1 above, reflects both the permeability and surface area of the glomerular membrane. The effect of glomerular pressure on *K*_*f*_ through changes in the glomerular surface area (hypertrophy) is modeled as:

15 $$\frac{d}{{dt}}\left( {\Delta SA} \right) = \left( {\Delta SA_{\mathrm{{max}}} - \Delta SA} \right) \ast \frac{{{\mathrm{GP}}\_{\mathrm{damage}}\_{\mathrm{effect}}}}{{\tau _{SA}}}$$

*ΔSA*_*max*_ is the maximum increase in glomerular surface area (SA; expressed as a percentage). *τ*_*SA*_ represents the time constant for the increase in surface area. *ΔSA*_*max*_ is fixed at 50%, the maximum increase observed in diabetic and/or nephrectomized rats, mice, and humans.^13,21,22^ The time constant *τ*_*SA*_ is set so that a steady-state is reached within a few weeks.

The effect of glomerular pressure on *K*_f_, through reductions in the glomerular permeability (glomerulosclerosis), is modeled as:

16 $$\frac{d}{{dt}}\left( {\Delta Perm} \right) = \left( {\Delta Perm_{max} - \Delta Perm} \right) \ast \frac{{{\mathrm{GP}}_{{\mathrm{damage}}_{{\mathrm{effect}}}}}}{{\tau _{perm}}}$$

*ΔPerm*_max_ is the maximal decrease in permeability (in percentage). *τ*_perm_ represents the time constant for the decrease in permeability. *ΔPerm*_*max*_ is assumed to be 100%, so that, eventually, permeability can decrease to zero.

The ultrafiltration coefficient *K*_*f*_ is then given by:

17 $$K_{\mathrm{f}} = K_{{\mathrm{f}},0} \ast \left( {1 + \Delta SA - \Delta {\mathrm{Perm}}} \right)$$

where *K*_*f,0*_ is the normal ultrafiltration coefficient in the healthy state.

The effect of glomerular pressure on nephron loss is modeled as:

17 $$\frac{d}{{dt}}\left( {\Delta {\mathrm{Nephrons}}} \right) = \left( {1 - \Delta {\mathrm{Nephrons}}} \right) \ast \frac{{{\mathrm{GP}}_{{\mathrm{damage}}_{{\mathrm{effect}}}}}}{{\tau _{{\mathrm{nephronLoss}}}}}$$

*Δ*Nephrons is the percentage of total nephrons lost, *τ*_nephronLoss_ represents the time constant for nephron loss.

Glomerular hypertension also damages to podocytes, causing them to leak protein. This is modeled as an increase in the sieving coefficient, in response to increased glomerular pressure:

18 $$\frac{d}{{dt}}\left( {\Delta K_{{\mathrm{albumin}}}} \right) = \frac{{{\mathrm{GP}}_{{\mathrm{damage}}_{{\mathrm{effect}}}}}}{{\tau _{{\mathrm{nephronLoss}}}}}$$

19 $$K_{{\mathrm{albumin}}} = K_{{\mathrm{albumin,0}}} + \Delta K_{{\mathrm{albumin}}}$$

*K*_albumin,0_ is the sieving coefficient under normal conditions, and τ_albumin_ is a time constant for the effect of glomerular pressure on sieving damage. Changes in albumin excretion in response to glomerular hypertension occur quickly, and thus this time constant is much larger than the other time constants (see Table 2). This is consistent with the fast changes in proteinuria observed with antihypertensive treatments, and in diseases such as preeclampsia.

### Model software and implementation

The model was implemented in the open-source programming software R 3.1.2, using the *RxODE* package ^41^.

## Electronic supplementary material

Supplement

## Data Availability

The authors declare that [the/all other] data supporting the findings of this study are available within the paper [and its supplementary information files], or in previous publications asvreferenced within the paper.

## Supplementary material

**Supplementary information:** accompanies the paper on the *npj Systems Biology and Applications* website (10.1038/s41540-018-0077-9).
