# Publisher Correction: Mathematical model of hemodynamic mechanisms and consequences of glomerular hypertension in diabetic mice

**DOI:** 10.1038/s41540-019-0081-8

**Published:** 2019-03-04

**Authors:** Hari Shankar Mahato, Christine Ahlstrom, Rasmus Jansson-Löfmark, Ulrika Johansson, Gabriel Helmlinger, K. Melissa Hallow

**Affiliations:** 1 School of Chemical, Materials, and Biomedical Engineering, University of Georgia, Athens, GA USA; 2 Drug Metabolism and Pharmacokinetics, Cardiovascular, Renal, and Metabolism, Innovative Medicines and Early Development Biotech Unit, AstraZeneca, Gothenburg, Sweden; 3 Bioscience Chronic Kidney Disease, Cardiovascular, Renal, and Metabolism Innovative Medicines and Early Development Biotech Unit, AstraZeneca, Gothenburg, Sweden; 4 Quantitative Clinical Pharmacology, Innovative Medicines and Early Development Biotech Unit, AstraZeneca, Waltham, MA USA

**Correction to:** *npj Systems Biology and Applications* 10.1038/s41540-018-0077-9, Published online 10 December 2018.

The original version of this Article had an incorrect Article number of 2, an incorrect Volume of 5 and an incorrect Publication year of 2019. These errors have now been corrected in the PDF and HTML versions of the Article.

