# Supplementary material for: Mathematical model of hemodynamic mechanisms and consequences of glomerular hypertension in diabetic mice
Source: NPJ Syst Biol Appl. 2018 Dec 10;4:41. doi: 10.1038/s41540-018-0077-9 (PMC6288095; doi:10.1038/s41540-018-0077-9)
Supplement: Supplementary file 1 — Supplement [file 41540_2018_77_MOESM1_ESM.pdf]

Supplemental Material

Contents:

Figures S1-S3

Detailed Methods: Full Model Equations

Model code

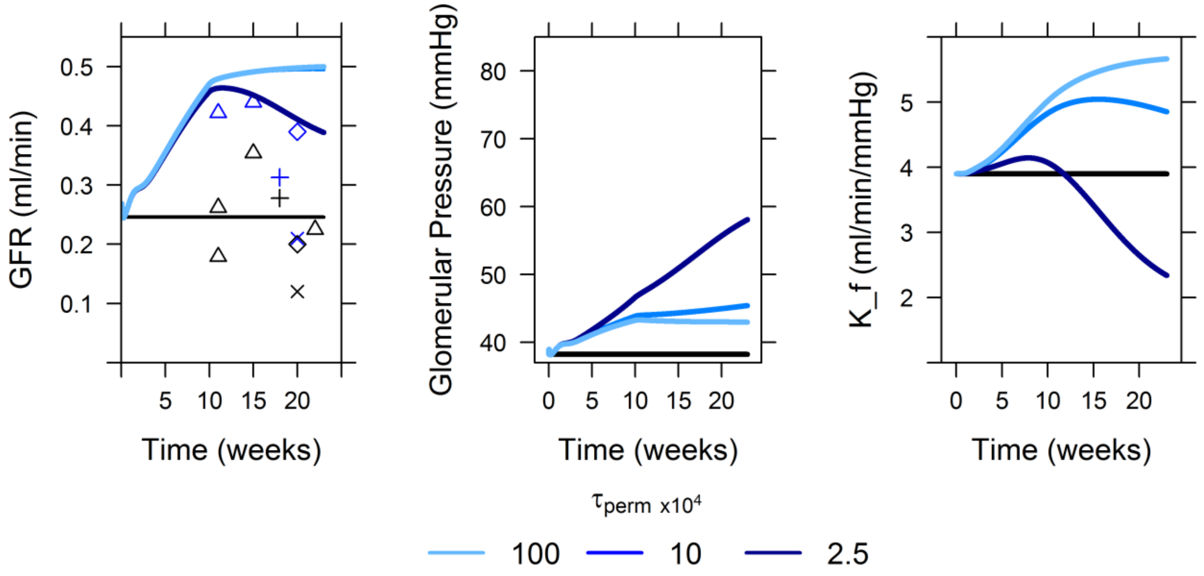

Figure S1. Sensitivity of simulation results to τ_{perm} – the time constant governing the effect glomerular pressure on glomerular membrane permeability. While Kf initially increases due to increasing surface area, glomerular pressure-induced reductions eventually cause Kf to decrease. As As τ_{perm} is reduced, Kf begins to fall more quickly, while glomerular pressure increasing further and GFR begins to decline earlier.

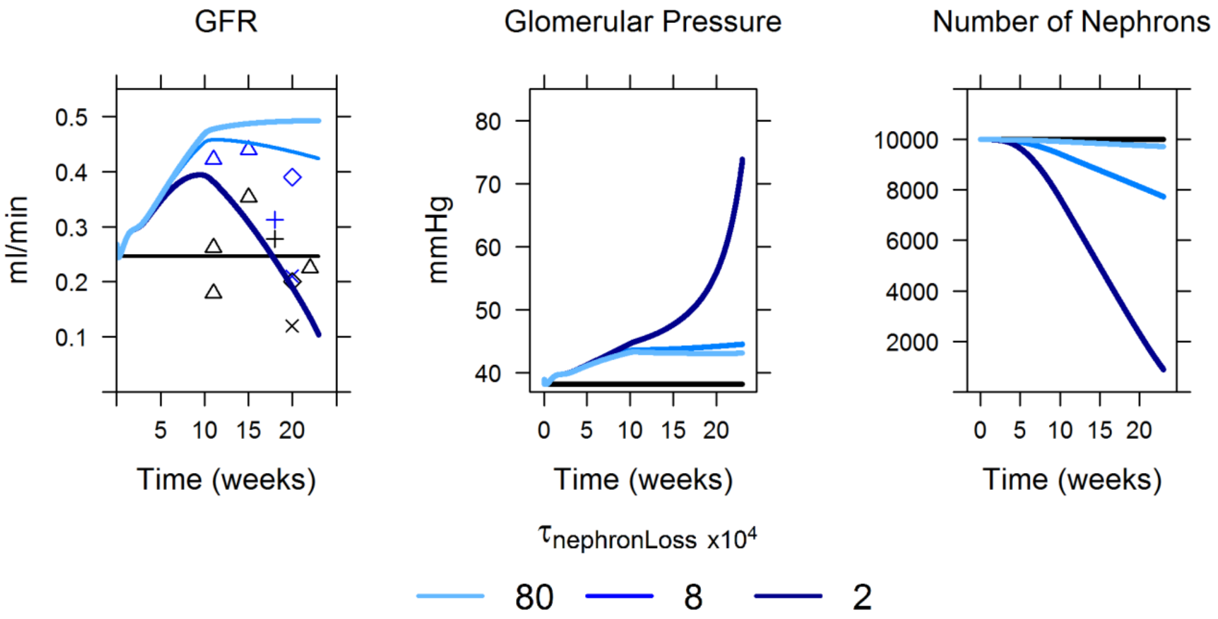

Figure S2. Sensitivity of simulation results to $\tau_{nephronLoss}$ – the time constant governing the effect glomerular pressure on nephron loss. As this time constant is decreased, nephrons are lost more rapidly, causing GFR to decrease and glomerular pressure to increase more quickly as wel.

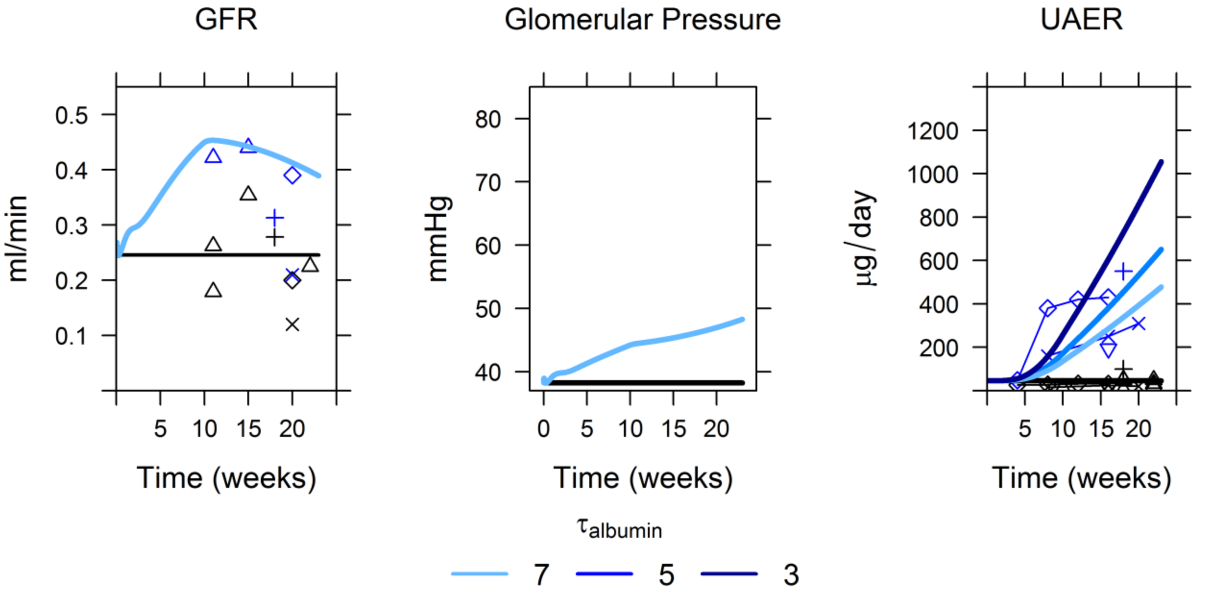

Figure S3. Sensitivity of simulation results to $\tau_{albumin}$ – the time constant governing the effect glomerular pressure on damage to the protein sieving membrane. This parameter does not affect GFR or glomerular pressure, but as this time constant gets smaller, the UAER response to increased glomerular pressure increases.

FULL MODEL EQUATIONS

Renal Vasculature

The glomeruli are modeled in parallel, and in series with the preafferent (interlobar, interlobular, and arcuate arterioles) and peritubular vasculature. Glomerular capillary resistance is assumed negligible. Thus, renal vascular resistance RVR is given by:

$$\mathrm{RVR} = \mathrm{R_{preaff}} + \frac{(R_{aa}+R_{ea})}{N_{nephrons}} + R_{peritubular} \qquad \text{Eq. A1}$$

R_{preaff} and $R_{peritubular}$ are lumped resistances describing the total resistance of preafferent and peritubular vasculatures, respectively, while R_{aa} and R_{ea} are the resistances of a single afferent or efferent arteriole, as determined from Pouiselle's law, based on the arteriole's diameter d, length L, and blood viscosity μ:

$$R_{aa} = \frac{128\mu L_{aa}}{\pi d_{aa}^4}; \qquad R_{ea} = \frac{128\mu L_{ea}}{\pi d_{ea}^4} \qquad \text{Eq. A2}$$

$N_{nephrons}$ is the number of nephrons. All nephrons are assumed identical, and the model does not account for spatial heterogeneity.

Renal blood flow (RBF) is a function of the pressure drop across the kidney and RVR, according to Ohm's law:

$$RBF = \frac{MAP-P_{renal-vein}}{RVR} + \frac{GFR\left(\frac{R_{ea}}{N_{nephrons}}\right)}{RVR} \qquad \text{Eq. A3}$$

Renal venous pressure ($P_{renal\text{-}vein}$) is treated as constant. The second term in this equation accounts for lower flow through the efferent arterioles due to GFR. As an approximation, all filtrate is assumed reabsorbed back into the peritubular capillaries, so that peritubular flow is the same as afferent flow.

P_{gc} is determined according to Ohm's law:

$$P_{gc} = MAP - RBF * (R_{preaff} + R_{aa}/N_{nephrons}) \qquad \text{Eq. A4}$$

Determination of MAP, P_{Bow} and $\pi_{go\text{-}avg}$ are described later.

Single nephron glomerular filtration rate (SNGFR) is defined according to Starling's equation, where K_f is the glomerular ultrafiltration coefficient, P_{gc} is glomerular capillary hydrostatic pressure, P_{Bow} is pressure in the Bowman's space, and $\pi_{go\text{-}avg}$ is average glomerular capillary oncotic pressure.

$$SNGFR = K_f\left(P_{gc} - P_{Bow} - \pi_{go-avg}\right) \qquad \text{Eq. A5}$$

The total GFR is then the SNGFR multiplied by the number of nephrons:

$$GFR = SNGFR * N_{nephrons} \qquad \text{Eq. A6}$$

Glucose filtration, reabsorption, and excretion

Glucose reabsorption occurs exclusively in the PT through Na^+ glucose cotransporters (SGLT). SGLT2 in the S1 and S2 segments of the PT reabsorbs 90% to 97% of filtered glucose, while SGLT1 in the S3 segment reabsorbs the remaining 3 to 10% [11, 51, 52]. As long as the filtered glucose load remains below the reabsorptive capacity of SGLT in the PT, glucose is nearly completely reabsorbed, and excreted glucose is negligible. However, at high plasma concentrations, filtered glucose can exceed the kidney's capacity for reabsorption, and the excess glucose is excreted. The plasma concentration at which filtered glucose exceeds the renal capacity for glucose reabsorption has been defined as the renal threshold for glucose excretion, RT_G. The renal capacity for glucose reabsorption, or $RC_{glucose}$, is given by:

$$RC_{glucose} = RT_G * SNGFR \qquad \text{Eq. 4}$$

The rate of glucose reabsorption is then:

$$\Phi_{glu,reabs} = \min(\Phi_{glu,filtered}, RC_{glucose}) \quad \text{Eq. 5}$$

Any non-reabsorbed glucose is then excreted, so that the rate of urinary glucose excretion (R_{UGE}) is:

$$R_{UGE} = \Phi_{glu,filtered} - \Phi_{glu,reabs} \quad \text{Eq. 6}$$

Na^+ filtration and reabsorption in the PT

Similarly to glucose, Na^+ is freely filtered across the glomerulus, so that the single nephron-filtered Na^+ load is given by:

$$\Phi_{Na,filtered} = SNGFR * C_{Na} \quad \text{Eq. 7}$$

where C_{Na} is the plasma Na^+ concentration.

Assuming that glucose reabsorption through SGLT1 is small compared to reabsorption through SGLT2, the rate of Na^+ reabsorption through SGLT2 is approximately equal to the rate of glucose reabsorption:

$$\Phi_{Na,reabs-SGLT2} = \Phi_{glu,reabs} \quad \text{Eq. 8}$$

Total PT Na^+ reabsorption is then given by:

$$\Phi_{Na,reabs-PT} = \Phi_{Na,filtered} * \eta_{pt,non-SGLT2} + \Phi_{Na,reabs-SGLT2} \quad \text{Eq. 9}$$

where $\eta_{pt,non-SGLT2}$ is the fractional rate of PT reabsorption through mechanisms other than SGLT2. Na^+ flow rate out of the PT is then:

$$\Phi_{Na,out-PT} = \Phi_{Na,filtered} - \Phi_{Na,reabs-PT} \quad \text{Eq. 10}$$

Na^+ reabsorption along the rest of the tubule is modeled as in [12].

Water Reabsorption along the tubule

Water reabsorption in the PT is isosmotic. Therefore, water leaving the PT and entering the loop of Henle is given by:

$$\Phi_{water,out-PT} = \Phi_{water,in-DCT} = SNGFR * \frac{\Phi_{osm,filtered}}{\Phi_{osm,out-PT}} \quad \text{Eq. A16}$$

where filtered osmolytes include both sodium and glucose:

$$\Phi_{osm,filtered} = 2 * \Phi_{Na,filtered} + \Phi_{glu,filtered} \qquad \text{Eq. c17}$$

$$\Phi_{osm,out-PT} = 2 * \Phi_{Na,out-PT} + \Phi_{glu,out-PT} \qquad \text{Eq. A18}$$

In the loop of Henle (LoH), water is reabsorbed in the water permeable descending LoH (DLH) due to the osmotic gradient created by actively pumping sodium out of the water-impermeable ascending limb (ALH). We have described modeling of this countercurrent mechanism previously(*23*), but only accounted for the osmotic effects of sodium. Here we adapt that description to include the osmotic activity of both sodium and glucose. The osmolality along the length of the DLH Osm_{DLH}, which is assumed in equilibrium with the osmolality in the surrounding interstitium Osm_{IS}, is given by:

$$Osm_{DLH}(x) = Osm_{IS}(x) = Osm_{DLH}(0)e^{\frac{R_{ALH}x}{\Phi_{water,in-DCT}Osm_{DLH}(0)}} \qquad \text{Eq. A19}$$

Where RALH is the rate of sodium reabsorption per unit length in the ascending loop of Henle (ALH), as described previously (our reference here). Water flow through the DLH is then given by:

$$\Phi_{water,DLH}(x) = \frac{\Phi_{water,DLH}(0)Osm_{DLH}(0)}{Osm_{DLH}(x)} \qquad \text{Eq. A20}$$

The ALH and the distal convoluted tubule (DCT) are modeled as impermeable to water, so that the flow through these segments equals the flow out of the DLH:

$$\Phi_{water,ALH}(x) = \Phi_{water,DCT}(x) = \Phi_{water,DLH}(L) \qquad \text{Eq. A21}$$

In the collecting duct (CD), water reabsorption is driven by the osmotic gradient between the CD tubular fluid and the interstitium, and is modulated by vasopressin:

$$\Phi_{\text{water,reabs}-\text{ CD}} = \eta_{vasopressin}\Phi_{\text{water,CD}}(0) * \left(1 - \frac{Osm_{CD}(L)}{Osm_{IS}(L)}\right) \quad \text{Eq. A22}$$

Where the osmolality in the CD $Osm_{CD}(L)$ accounts for sodium reabsorbed in the collecting duct:

$$Osm_{CD}(L) = \frac{\Phi_{\text{osm,cd}}(0) - 2 * \left(\Phi_{\text{Na,cd}}(0) - \Phi_{\text{Na,cd}}(L)\right)}{\Phi_{\text{water,CD}}(0)} \quad \text{Eq. A23}$$

The effect of vasopressin on water reabsorption is modeled as a sigmoidal relationship, such that $\eta_{vasopressin}$ is one and water flows freely across the osmotic gradient when vasopressin levels are very high, and $\eta_{vasopressin}$ is zero and no water is reabsorbed when vasopressin in absent.

$$\eta_{vasopressin} = \frac{vasopressin}{Km_{vp} + vasopressin} \quad \text{Eq. A24}$$

Then, single nephron water excretion rate is given by:

$$\Phi_{\text{water,CD}}(L) = \Phi_{\text{water,CD}}(0) - \Phi_{\text{water,reabs}-\text{ CD}} \quad \text{Eq. A25}$$

And urine flow rate is then:

$$\Phi_{\text{urine}} = \text{SNGFR} * \Phi_{\text{water,CD}}(L) \quad \text{Eq. A26}$$

Peripheral Sodium Storage

We incorporated that three compartment model of volume homeostasis into the renal physiology model, to allow evaluation of the potential role of peripheral sodium storage in the renal response to dapagliflozin. Sodium and water are assumed to move freely between the blood and interstitial fluid. Water and sodium intake rates were assumed constant. Then blood volume (BV) and blood sodium (Nablood) are the balance between intake and excretion of water and sodium respectively, and the intercompartmental transfer.

$$\frac{d}{dt}(BV) = Water_{in} - Water_{out} + Q_{water}([Na]_{blood} - [Na]_{IF})\text{Eq.} \qquad \text{Eq. A27}$$

$$\frac{d}{dt}(Na_{blood}) = \Phi_{\text{Na,intake}} - \Phi_{\text{Na,excretion}} + Q_{Na}([Na]_{IF} - [Na]_{blood}) \qquad \text{Eq. A28}$$

Sodium concentrations in the blood and interstitial compartments are assumed to equilibrate quickly. Change in interstitial fluid volume (IFV) is a function of intercompartmental water transfer.

$$\frac{d}{dt}(IFV) = Q_{water}([Na]_{IF} - [Na]_{blood}) \qquad \text{Eq. A29}$$

When interstitial sodium concentration $[Na]_{IF}$ exceeds the normal equilibrium level $[Na]_{IF,ref}$, Na+ moves out of the interstitium and is sequestered in the peripheral Na+ compartment, at a rate of $\Phi_{Na,stored}$, where it is osmotically inactive. Thus, the change interstitial fluid sodium depends on intercompartmental transfer and peripheral storage. Sodium cannot be stored indefinitely, and thus there is a limit Nastored,max on how much sodium can be stored. The peripheral sodium compartment can be effectively removed from the model by setting $Q_{Na,stored}$ to zero.

$$\Phi_{\text{Na,stored}} = Q_{Na,stored} * \frac{(Na_{stored,max} - Na_{stored})}{Na_{stored,max}}\left([Na]_{JF} - [Na]_{IF,ref}\right) \qquad \text{Eq. A30}$$

$$\frac{d}{dt}(Na_{stored}) = \Phi_{\text{Na,stored}} \qquad \text{Eq. A31}$$

$$\frac{d}{dt}(Na_{IF}) = Q_{Na}([Na]_{blood} - [Na]_{IF}) - \Phi_{\text{Na,stored}} \qquad \text{Eq. A32}$$

Blood and IF sodium concentrations are then given by:

$$[Na]_{blood} = \frac{Na_{blood}}{BV} \qquad \text{Eq. A33}$$

$$[Na]_{IF} = \frac{Na_{ecf}}{IFV} \qquad \text{Eq. A34}$$

Cardiovascular Function

The mean cardiac filling pressure (P_{mf}) is a function of blood volume and venous compliance c_{venous}.

$$P_{mf} = P_{mf,0} + \frac{V_b - V_{b,0}}{c_{venous}} \qquad \text{Eq. A35}$$

Cardiac output (CO) is mean filling pressure divided by resistance to venous return (R_{vr}).

$$CO = \frac{P_{mf}}{R_{vr}} \qquad \text{Eq. A36}$$

Total peripheral resistance (TPR) is determined by treating systemic and renal vasculatures as parallel resistances, in series with venous resistance:

$$TPR = SVR * \frac{RVR}{SVR + RVR} + R_{venous} \qquad \text{Eq. A37}$$

According to Ohm's law, mean arterial pressure (MAP) is then:

$$MAP = CO * TPR \qquad \text{Eq. A38}$$

Modeling Tubular Hydrostatic Pressure

Hydrostatic pressure in the Bowman's space is a key factor affecting GFR, and this pressure is influenced by both morphology and flow rates through the tubule. Changes in Na and water reabsorption along the nephron, which can occur either due to disease or treatments, can alter GFR by altering tubular pressures. Thus dynamically modeling tubular pressures can be critical to understanding GFR changes.

Adapting from Jensen et al(16), tubular flow rates described in the main text can be used to determine tubular pressure. The change in intratubular pressure dP^* over a length of tubule dx can be defined according to Poiseuille's law as:

$$dP^* = -\frac{128\eta}{\pi D^4}\Phi_{water}(x)dx \qquad \text{Eq. A39}$$

Eq. 22 describes the relationship between transtubular pressure P and tubular diameter D, where D_c is the diameter at control pressure P_c, and β is the exponent of tubular distensibility (16).

$$\frac{D}{D_c} = \left(\frac{P}{P_c}\right)^{\beta} \qquad \text{Eq. A40}$$

Substituting and assuming uniform interstitial pressure throughout the kidney, we obtain:

$$dP = -\frac{128\eta}{\pi D_c^4}\left(\frac{P_c}{P}\right)^{4\beta}\Phi_{water}(x)\,dx \qquad \text{Eq A41}$$

Integrating over a tubule segment length, we obtain inlet pressure as a function of the outlet pressure and the flow rate:

$$P_{in} = \left[P_{out}^{4\beta+1} + \frac{(4\beta+1)128\eta P_c^{4\beta}}{\pi D_c^4}\int_0^L \Phi_{water}(x)dx\right]^{\frac{1}{4\beta+1}} \qquad \text{Eq A42}$$

The pressure calculated at the inlet to the PT is used as P_{Bow} in Eq. 4 above.

Because the diameter of the CNT/CD changes as nephrons coalescence, calculating pressure along this segment is challenging. Under normal conditions, pressure drops 5-7mmHg across the CNT/CD (16). Thus, an effective control diameter was calculated to give this degree of pressure drop under baseline conditions.

Modeling Glomerular Capillary Oncotic Pressure

The glomerular capillary oncotic pressure is calculated using the Landis Pappenheimer equation, where C_{prot} is the concentration of protein at the point of interest.

$$\pi = 1.629 * C_{prot} + 0.2935 * C_{prot}^2 \quad \text{Eq. A43}$$

Plasma protein ($C_{prot\text{-}plasma}$) is assumed constant. Protein concentration at the distal end of the glomerulus ($C_{prot\text{-}glom\text{-}out}$) is determined as:

$$C_{prot-glom-out} = C_{prot-plasma} * \frac{RBF}{RBF-GFR} \quad \text{Eq. A44}$$

Protein concentration is assumed to be varying linearly along the capillary length, and thus the oncotic pressure π_{go-avg} is calculated using the average of the plasma protein concentration and protein concentration at the distal end of the glomerulus.

The model does not account for filtration equilibrium, which occurs in some species.

Modeling Regulatory Mechanisms

Multiple control mechanisms act on the system to allow simultaneous control of C_{na}, CO, MAP, glomerular pressure, and RBF. For each control mechanism, the feedback signal μ is modeled by one of two functional forms. The choice of functional form is determined by whether a steady state error is allowed in the controlled variable X. When a steady state error is not allowed (i.e. X always eventually returns to the setpoint X_0), the effect is defined by a proportional-integral (PI) controller. The initial feedback signal is

proportional to the magnitude of the error ($X-X_0$), with gain G. But the feedback continues to grow over time as long as any error exists, until the error returns to zero. The integral gain K_i determines the speed of return to steady-state.

$$\mu = 1 + G * ((X - X_0) + K_i * \int (X - X_0)dt) \qquad \text{Eq. 45}$$

All other mechanisms, for which the controlled variable can deviate from the setpoint at steady-state, are described by a logistic equation that produces a saturating response characteristic of biological signals:

$$\mu = 1 + S * \left(\frac{1}{1+\exp\left(\frac{X-X_0}{m}\right)} - 0.5 \right) \qquad \text{Eq. 46}$$

Here, m defines the slope of the response around the operating point, and S is the maximal response as X goes to $\pm\infty$.

Control of plasma Na concentration by vasopressin

Changes in plasma osmolality are sensed via osmoreceptors, stimulating vasopressin secretion, which exerts control of water reabsorption in the CNT/CD. To insure that blood sodium concentration C_{Na} is maintained at its setpoint $C_{Na,0}$ at steady state, this process is modeled by a PI controller:

$$\mu_{vasopressin} = 1 + G_{Na-vp} * \left(C_{Na} + K_{i-vp} * \int \left(C_{Na} - C_{Na,0}\right)dt\right) \qquad \text{Eq. 47}$$

The parameters G_{Na-vp} and K_{i-vp} are gains of proportional and integral control, respectively.

Tubular Pressure Natriuresis

For homeostasis, Na excretion over the long-term must exactly match Na intake (the principle of Na balance). Any steady-state Na imbalance would lead to continuous volume retention or loss– an untenable situation. Pressure-natriuresis(2), wherein changes in renal perfusion pressure (RPP) induce changes in Na excretion, insures that Na balance is maintained. It may be partially achieved through neurohumoral

mechanisms including the RAAS, but there is also an intrinsic pressure-mediated effect on tubular Na reabsorption, where renal interstitial hydrostatic pressure (RIHP) is believed to be the driving signal. RIHP is a function of peritubular capillary pressure, and is calculated according to Ohm's law:

$$P_{peritubular} = MAP - RBF * \left(R_{preaff} + \frac{R_{aff}+R_{eff}}{N_{nephrons}}\right) \qquad \text{Eq. 48}$$

As a simplification, we assume an increase in peritubular pressure will generate a proportional increase in RIHP. Since the kidney is encapsulated, we assume interstitial pressure equilibrates and changes in one region are transduced across the kidney. The relationship between RIHP and fractional Na reabsorption rate of each tubular segment is then modeled as:

$$\eta_{i-sodreab} = \eta_{i-sodreab,0} * \left(1 + S_{P-N,i} * \left(\frac{1}{1+\exp(RIHP-RIHP_0)} - 0.5\right)\right) \qquad \text{Eq. 49}$$

where i = PT, LoH, DCT, or CNT/CD. $\eta_{i-sodreab,0}$ is the nominal fractional rate of reabsorption for that tubule segment. $RIHP_0$ defines the setpoint pressure and is determined from RIHP at baseline for normal Na intake. $S_{P-N,i}$ defines the maximal signal as RIHP goes to ∞.

Control of Cardiac Output

CO, which describes total blood flow to body tissues, returns to normal over days to weeks following a perturbation (38). CO regulation is a complex phenomenon that occurs over multiple time scales, but we focus only on long-term control (days to weeks), which is thought to be achieved through whole-body autoregulation - the intrinsic ability of organs to adjust their resistance to maintain constant flow(38). The total effect of local autoregulation of all organs is that TPR is adjusted to maintain CO at a constant resting level. The feedback between CO and TPR is modeled with a PI controller, such that CO is controlled to its steady-state setpoint CO_0.

$$\mathrm{TPR} = \mathrm{TPR}_0 * \left(1 + \mathrm{G}_{\mathrm{CO-tpr}} * \left(\mathrm{CO} + \mathrm{K}_{\mathrm{i-tpr}} * \int(\mathrm{CO} - \mathrm{CO}_0)\mathrm{dt}\right)\right) \qquad \text{Eq. 50}$$

Control of Macula Densa Sodium Concentration by Tubuloglomerular Feedback

Tubuloglomerular feedback (TGF) helps stabilize tubular flow by sensing Na concentration in the the macula densa, which sits between the LoH and DCT, and providing a feedback signal to inversely change afferent arteriole diameter. The TGF effect is defined as:

$$\mu_{\mathrm{TGF}} = 1 + \mathrm{S}_{\mathrm{TGF}} * \left(\frac{1}{1+\exp\left(\frac{\mathrm{C}_{Na,\mathrm{MD},0} - \mathrm{C}_{\mathrm{Na,MD}}}{\mathrm{m}_{\mathrm{TGF}}}\right)} - 0.5\right) \qquad \text{Eq. A51}$$

The basal afferent arteriole resistance Raa is then multiplied by μ_{TGF} to obtain the ambient afferent arteriolar resistance. The setpoint $C_{Na,MD,0}$ is the Na concentration out of the LoH and into the DCT in the baseline state at normal Na intake.

Myogenic Autoregulation of Glomerular Pressure

Glomerular hydrostatic pressure is normally tightly autoregulated, and changes very little in response to large changes in blood pressure. This autoregulation is in part through myogenic autoregulation of the preglomerular arterioles. While the pressure drop and thus myogenic response varies along the arteriole length, we make the simplifying assumption that the preafferent vasculature responds to control pressure at the distal end.

$$\mu_{autoreg} = 1 + S_{autoreg} * \left(\frac{1}{1+\exp\left(\frac{P_{preafferent} - P_{preafferent,0}}{m_{autoreg}}\right)} - 0.5\right) \qquad \text{Eq. A52}$$

Pressure at the distal end of the preafferent vasculature is given by:

$$P_{preafferent} = MAP - RBF * R_{preaff} \qquad \text{Eq. A53}$$

The basal preafferent arteriole resistance R_{preaff} is then multiplied by $\mu_{autoreg}$ to obtain the ambient preafferent arteriolar resistance.

Renin-Angiotensin-Aldosterone System Submodel

Renin is secreted at a nominal rate $SEC_{ren,0}$ modulated by macula densa sodium flow, as well as by a strong negative feedback from Angiotensin bound to the AT1 receptor.

$$SEC_{renin} = \mu_{md-renin} * \mu_{AT1} * SEC_{renin,0} \quad \text{Eq. A54}$$

The macula densa releases renin in response to reduced sodium flow:

$$\mu_{md-renin} = e^{-A_{md-ren}(\phi_{Na,md} - \phi_{Na,md,0})} \quad \text{Eq. A55}$$

We have found that the inhibitory effect of AT1-bound AngII on renin secretion can be well described by the following relationship:

$$\mu_{AT1} = 10^{A_{AT1,ren} * \log 10(AT1-bound\_AngII - AT1-bound\_AngII,0)} \quad \text{Eq. A56}$$

Plasma renin concentration (PRC) is then given by:

$$\frac{d(PRC)}{dt} = SEC_{renin} - K_{d,renin} * PRC \quad \text{Eq. A57}$$

Where ~~Krenin~~ $K_{d,renin}$ is the renin degradation rate. PRA can be related to PRC by the conversion factor 0.06 (ng/ml/hr)/(pg/ml).

Angiotensin I is formed by PRA, assuming that its precursor angiotensinogen is available in excess and the plasma renin activity (PRA) is the rate-limiting step. AngI is also converted to AngII by the enzymes ACE and chymase, and is degraded at a rate of $K_{d,AngI}$.

$$\frac{d(AngI)}{dt} = PRA - (ACE + Chymase) * AngI - K_{d,AngI} AngI \quad \text{Eq. A58}$$

Angiotensin II is formed from the action of ACE and chymase on AngI, can be eliminated by binding to either the AT1 or AT2 receptors at the rate C_{AT1} and C_{AT2} respective, and is degraded at a rate of Kd,AngII.

$$\frac{d(AngII)}{dt} = (ACE + \ Chymase) * AngI - (C_{AT1} + C_{AT2}) * AngII - K_{d,AngII}AngII \quad \text{Eq. A59}$$

The complex of Angiotensin II bound to the AT1 receptor is the physiologically active entity within the pathway, and is given by:

$$\frac{d\left(AT1_{bound_{AngII}}\right)}{dt} = (C_{AT1}) * AngII - K_{d,AT1}AT1\_bound\_AngII \quad \text{Eq. A60}$$

AT1-bound AngII has multiple physiologic effects, including constriction of the efferent, as well and preglomerular, afferent, and systemic vasculature, sodium retention in the PT, and aldosterone secretion. Each effect is modeled as:

$$\mu_{AT1,i} = 1 + S_{AT1,i} * \left(\frac{1}{1+\exp\left(\frac{AT1-bound_{AngII_0} - AT1-bound\_AngII}{m_{AT1,i}}\right)} - 0.5 \right) \quad \text{Eq. A61}$$

where i represents the effect on efferent, afferent, preafferent, or systemic resistance, PT sodium reabsorption, or aldosterone secretion.

Aldosterone is the second physiologically active entity in the RAAS pathway, acting by binding to mineralocorticoid receptors (MR) in the CNT/CD and DCT to stimulate sodium reabsorption. MR-bound aldosterone is modeled as the nominal concentration Aldo,0 modulated by the effect of AT1-bound AngII, and the normalized availability of MR receptors (1 in the absence of an MR antagonist).

$$MR - bound\_Aldo = Aldo_0 * \mu_{AT1} * MR \quad \text{Eq. A62}$$

The effects of MR-bound aldosterone on CNT/CD and DCT sodium reabsorption are modeled as:

$$\mu_{aldo,i} = 1 + S_{aldo,i} * \left(\frac{1}{1+exp\left(\frac{MR-bound\ Aldo_0 - MR-bound\ Aldo}{m_{aldo,i}}\right)} - 0.5 \right) \quad \text{Eq. A63}$$

Where i is the CNT/CD or DCT.

Calculation of Dependent Model Parameter Values Required for Steady State

For some parameters, although a range may be established from the literature, it makes more sense to calculate the parameter based on values or setpoints for other parameters (while ensuring that the calculated value falls within the reported range). For instance, rather than specifying nominal systemic vascular resistance (SVR0), we can calculate what it must be to give the expected baseline cardiac output and MAP:

$$\mathrm{SVR_0} = \frac{\mathrm{MAP_0}}{\mathrm{CO_0}} \quad \text{Eq. A64}$$

Similarly, nominal renal vascular resistance (RVR_0) can be calculated based on normal MAP and RBF values:

$$\mathrm{RVR_0} = \frac{\mathrm{MAP_0} - \mathrm{P_{renal-vein}} + \mathrm{GFR_0}\left(\frac{\mathrm{R_{ea}}}{\mathrm{Nnephrons}}\right)}{\mathrm{RBF_0}} \quad \text{Eq. A65}$$

Both peritubular vascular resistance ($R_{peritubular,0}$) and CNT/CD fractional reabsorption ($\eta_{CNT/CD,0}$) are difficult to measure precisely, and a wide range of values have been reported in the literature, primarily from non-human sources. For these parameters, also, we can instead calculate what their values must be to give specified steady-state values for more easily measureable parameters. $R_{peritubular,0}$ can be calculated based on the RVR_0 calculated as given above, and reported values for afferent, efferent, and preglomerular resistances, which are known with greater certainty:

$$\mathrm{R_{peritubular}} = (\mathrm{RVR_0} - \mathrm{R_{preaff,0}}) * \mathrm{N_{nephrons}} - \mathrm{R_{aff,0}} - \mathrm{R_{eff,0}} \quad \text{Eq. A66}$$

Similarly, $\eta_{CNT/CD,0}$ can be calculated for a given equilibrium GFR, plasma Na concentration, and Na intake, as well as upstream fractional Na reabsorption rates, based on the fact that at equilibrium, Na excretion must equal Na intake (Φ_{sodin}). Na flow out of each segment can be calculated as:

$$\Phi_{out,pt,0} = GFR_0 * C_{Na,0} * (1 - \eta_{pt}) \quad \text{Eq. A67}$$

$$\Phi_{out,loh,0} = \Phi_{out,pt,0}(1 - \eta_{loh}) \quad \text{Eq. A68}$$

$$\Phi_{out,dct,0} = \Phi_{out,loh,0}(1 - \eta_{dct}) \quad \text{Eq. A69}$$

Na flow out of the CNT/CD must equal sodium intake:

$$\Phi_{out,cd,0} = \Phi_{out,dct,0}(1 - \eta_{cnt/cd,0}) = \Phi_{sodin} \quad \text{Eq. A70}$$

Then, solving for ηcnt/cd,0 gives:

$$\eta_{cnt/cd,0} = 1 - \Phi_{sodin}/\Phi_{out,dct,0} \quad \text{Eq. A71}$$

There is substantial variability in experimental measurements of fractional Na reabsorption rates for each tubular segments. PT fractional reabsorption has been reported across the range of 40-78% (40-44). Under normal flow conditions, LoH fractional Na reabsorption has been reported from 85-93%(23, 45). For this analysis, fractional Na reabsorption rates in the PT, LoH, and DCT were set to 0.70, 0.88, and 0.5, respectively, and CNT/CD fractional reabsorption rate was calculated to be 0.827.

Model Code

The model was implemented in a free open-source programming software (R 3.1.2). It utilizes the RxODE package.

```
#In this simplified model, almost all neurohormonal effects have been removed

#An effect of RSNA on afferent resistance remains as this is necessary for model stability

#Autoregulatory mechanisms remain, including TGF, whole body autoregulation, myogenic
autoregulation




############################## Define model structure #########################

ode <- "


  number_of_functional_nephrons = no_of_kidney*baseline_nephrons*(1 -
0.3*kf_permeability_increase);

  number_of_functional_tubules = number_of_functional_nephrons*(1-fraction_nephrons_lost);

  glomerular_hydrostatic_conductance_Kf = nom_Kf*(1+kf_surface_increase-kf_permeability_increase);


  ###Systemic Hemodynamics


  tissue_autoregulation_signal =
max(0.1,1+tissue_autoreg_scale*((Kp_CO/CO_scale_species)*(cardiac_output_delayed -
CO_nom)+(Ki_CO/CO_scale_species)*CO_error));

  systemic_arterial_resistance = tissue_autoregulation_signal*(nom_systemic_arterial_resistance);

  total_peripheral_resistance = systemic_arterial_resistance + R_venous;

  resistance_to_venous_return = ((8 * R_venous + systemic_arterial_resistance) / 31);
```

blood_volume_L = BV_scale_species*(4.56+2.43/(1+exp(-((extracellular_fluid_volume/ECF_scale_species-18.1))*0.474)));

mean_filling_pressure = (((BV_filling_pressure_slope/BV_scale_species) * blood_volume_L - reference_BV_mean_filling_pressure));

venous_return = ((mean_filling_pressure) / resistance_to_venous_return);

cardiac_output = venous_return;

mean_arterial_pressure_MAP = (cardiac_output * total_peripheral_resistance);

###Blood pressure effect on renal sympathetic nerve activity

map_rsna_int = 1 - map_rsna_scale/2;

MAP_effect_on_rsna = map_rsna_int + map_rsna_scale / (1 + exp((mean_arterial_pressure_MAP - nominal_map_setpoint) / map_rsna_slope));

renal_sympathetic_nerve_activity = nom_rsna*MAP_effect_on_rsna ;

Renal vasculature hemodynamimcs

#Preafferent arterioles

preaff_arteriole_signal_multiplier = preafferent_pressure_autoreg_signal;

preaff_arteriole_adjusted_signal_multiplier = (1/(1+exp(preaff_signal_nonlin_scale*(preaff_arteriole_signal_multiplier-1)))+0.5);

nom_effective_preaff_diameter = (nom_preafferent_arteriole_resistance*(1-preafferent_resistance_decrease))^(-0.25);

```
  preaff_diameter =
(nom_effective_preaff_diameter)+preaff_diameter_range*(preaff_arteriole_adjusted_signal_multiplier-
1);

  preafferent_arteriole_resistance = 1/(preaff_diameter ^4);


  # Afferent arterioles

  # (A feedback of rsna on afferent resistance is necessary for model stability)

  rsna_aff_intercept = 1-rsna_aff_scale/2;

  rsna_effect_on_aff_resistance = max(0.25,rsna_aff_intercept + rsna_aff_scale/(1+exp((1-
renal_sympathetic_nerve_activity)/rsna_aff_slope)));


  # total effect of all signals affecting afferent resistance

  afferent_arteriole_signal_multiplier = rsna_effect_on_aff_resistance *
tubulo_glomerular_feedback_effect *glomerular_pressure_autoreg_signal;

  afferent_arteriole_adjusted_signal_multiplier =
(1/(1+exp(afferent_signal_nonlin_scale*(afferent_arteriole_signal_multiplier-1)))+0.5);

  afferent_diameter = nom_afferent_diameter*((1-preafferent_resistance_decrease)^(-
0.25))+afferent_diameter_range*(afferent_arteriole_adjusted_signal_multiplier-1);

  afferent_arteriole_resistance = L_m3*viscosity_length_constant/(afferent_diameter^4);


  # Efferent arterioles

  efferent_arteriole_signal_multiplier =1;

  efferent_arteriole_adjusted_signal_multiplier =
1/(1+exp(efferent_signal_nonlin_scale*(efferent_arteriole_signal_multiplier-1)))+0.5;

  efferent_diameter =
nom_efferent_diameter+efferent_diameter_range*(efferent_arteriole_adjusted_signal_multiplier-1);

  efferent_arteriole_resistance = L_m3*viscosity_length_constant/(efferent_diameter^4);


  #Peritubular
```

RBF_autoreg_int = 1 - RBF_autoreg_scale/2;

peritubular_autoreg_signal = RBF_autoreg_int + RBF_autoreg_scale/(1+exp((nom_renal_blood_flow_L_min - renal_blood_flow_L_min_delayed)/RBF_autoreg_steepness));

autoregulated_peritubular_resistance = peritubular_autoreg_signal*nom_peritubular_resistance;

#Total renal vascular resistance

renal_vascular_resistance = (preafferent_arteriole_resistance + (afferent_arteriole_resistance + efferent_arteriole_resistance) / (number_of_functional_nephrons/no_of_kidney) + autoregulated_peritubular_resistance);

#Renal blood flow

renal_blood_flow_L_min = ((pre_renal_blood_pressure - P_venous) / renal_vascular_resistance);

renal_blood_flow_ml_hr = renal_blood_flow_L_min * 1000 * 60;

#Renal pressures

preafferent_pressure = mean_arterial_pressure_MAP - renal_blood_flow_L_min*preafferent_arteriole_resistance;

glomerular_pressure = (pre_renal_blood_pressure - renal_blood_flow_L_min * (preafferent_arteriole_resistance + afferent_arteriole_resistance / (number_of_functional_nephrons/no_of_kidney)));

postglomerular_pressure = (pre_renal_blood_pressure - renal_blood_flow_L_min * (preafferent_arteriole_resistance + (afferent_arteriole_resistance+efferent_arteriole_resistance) / (number_of_functional_nephrons/no_of_kidney)));

#Autoregulatory signals to the preafferent and afferent arterioles are calculated based on teh preafferent ang glomerular pressures

gp_autoreg_int = 1 - gp_autoreg_scale/2;

preaff_autoreg_int = 1 - preaff_autoreg_scale/2;

preafferent_pressure_autoreg_function = preaff_autoreg_int+preaff_autoreg_scale/(1+exp((nom_preafferent_pressure - preafferent_pressure)/myogenic_steepness));

glomerular_pressure_autoreg_function = gp_autoreg_int+gp_autoreg_scale/(1+exp((nom_glomerular_pressure - glomerular_pressure)/myogenic_steepness));

#preafferent_change = (max_preafferent_decrease - preafferent_resistance_decrease)*max(mean_arterial_pressure_MAP - nominal_map_setpoint - 10,0)/T_preafferent;

preafferent_change = (max_preafferent_decrease - preafferent_resistance_decrease)*max(glucose_concentration - 120,0)/T_preafferent;

Renal filtration

net_filtration_pressure = glomerular_pressure - oncotic_pressure_difference - P_bowmans;

SNGFR_nL_min = glomerular_hydrostatic_conductance_Kf * (glomerular_pressure - oncotic_pressure_difference - P_bowmans);

SNGFR_mL_min = SNGFR_nL_min*mL_nL;

GFR = (SNGFR_nL_min / 1000 / 1000000 * number_of_functional_tubules);

GFR_ml_min = GFR * 1000;

#Glomerular Capillary oncotic pressure

```
  Oncotic_pressure_in = 
1.629*plasma_protein_concentration+0.2935*(plasma_protein_concentration^2);

  SNRBF_nl_min = 1e6*1000*renal_blood_flow_L_min/number_of_functional_nephrons;

  plasma_protein_concentration_out = SNRBF_nl_min*plasma_protein_concentration/(SNRBF_nl_min-
SNGFR_nL_min);

  Oncotic_pressure_out = 
1.629*plasma_protein_concentration_out+0.2935*(plasma_protein_concentration_out^2);

  oncotic_pressure_avg = (Oncotic_pressure_in+Oncotic_pressure_out)/2;


      serum_creatinine_concentration = serum_creatinine/blood_volume_L;

      creatinine_clearance_rate = GFR_ml_min * dl_ml * serum_creatinine_concentration; #Units: 
mg/min


################################ Added Recently 
######################################################################




#Allow glucose concentration and sodium intake to increase over time

glucose_concentration_change=max(maximum_glucose_concentration - glucose_concentration, 0)/
T_glucose;

Na_intake_rate_change = max(maximum_Na_intake - Na_intake, 0)/T_Na;


###Glucose filtation

filtered_glucose = GFR*glucose_concentration*10; #units mg/min

renal_threshold_glucose_reabsorption = nom_renal_threshold_glucose_reabsorption*SGLT2_inhibition; 
#mg/dl

max_glucose_reabsorption = renal_threshold_glucose_reabsorption*GFR*10; #units mg/min

glucose_reabsorption = min(max_glucose_reabsorption, filtered_glucose);
```

rate_urinary_glucose_excretion =(max(0, filtered_glucose - max_glucose_reabsorption)*60*24)/1000;#gm/day

SGLT2_Na_reabsorption = glucose_reabsorption*glucose_mg_mmol; #Assumes 1:1 molar reabsorption. Ignores SGLT1

########################### End ###

###Whole body fluid adn electrolye balance, and vasopressin secretion

#Plasma sodium concentration

Na_concentration = (sodium_amount / extracellular_fluid_volume);

#Vasopressin secretion is determined by a proportional-integral controller of sodium concentration

Na_water_controller = 1+Na_controller_gain*(Kp_VP*(Na_concentration - ref_Na_concentration)+Ki_VP*Na_concentration_error);

normalized_vasopressin_concentration = 1 + Na_water_controller;

vasopressin_concentration = nominal_vasopressin_conc * normalized_vasopressin_concentration;

#Water intake rate

water_intake_vasopressin_int = 1-water_intake_vasopressin_scale/2;

water_intake = water_intake_species_scale*(nom_water_intake/60/24)*(water_intake_vasopressin_int + water_intake_vasopressin_scale/(1+exp((normalized_vasopressin_concentration-1)/water_intake_vasopressin_slope)));

daily_water_intake = (water_intake * 24 * 60);

###Filtered sodium load

SN_filtered_Na_load = (SNGFR_nL_min / 1000 / 1000000)*Na_concentration;

filtered_Na_load = SN_filtered_Na_load*number_of_functional_tubules;

###Tubular sodium reabsorption

#Tubular pressure-natriuresis mechanisms - signals   are a function of renal interstitial hydrostatic pressure

pressure_natriuresis_PT_int = 1 - pressure_natriuresis_PT_scale/2;

pressure_natriuresis_PT_effect = max(0.001,pressure_natriuresis_PT_int + pressure_natriuresis_PT_scale / (1 + exp((postglomerular_pressure- RIHP0) / pressure_natriuresis_PT_slope)));

pressure_natriuresis_LoH_int = 1 - pressure_natriuresis_LoH_scale/2;

pressure_natriuresis_LoH_effect = max(0.001,pressure_natriuresis_LoH_int + pressure_natriuresis_LoH_scale / (1 + exp((postglomerular_pressure - RIHP0) / pressure_natriuresis_LoH_slope)));

pressure_natriuresis_DT_int = 1 - pressure_natriuresis_DT_scale/2;

pressure_natriuresis_DT_effect = max(0.001,pressure_natriuresis_DT_int + pressure_natriuresis_DT_scale / (1 + exp((postglomerular_pressure - RIHP0) / pressure_natriuresis_DT_slope)));

pressure_natriuresis_CD_magnitude = max(0,pressure_natriuresis_CD_scale);

pressure_natriuresis_CD_int = 1 - pressure_natriuresis_CD_magnitude/2;

```
  pressure_natriuresis_CD_effect = max(0.001,pressure_natriuresis_CD_int + pressure_natriuresis_CD_magnitude/ (1 + exp((postglomerular_pressure - RIHP0) / pressure_natriuresis_CD_slope)));



  #Proximal tubule sodium reabsorption

 # e_pt_sodreab = nominal_pt_na_reabsorption * pressure_natriuresis_PT_effect;  #PT fractional rate of reabsorption


######################### Added recently ############################# Start

e_pt_sodreab = nominal_pt_na_reabsorption * pressure_natriuresis_PT_effect;  #PT fractional rate of reabsorption


##Proximal tubule reabsorption and outflow

SN_proximal_tubule_reabsorption = min(SN_filtered_Na_load,renal_threshold_Na_reabs)*(e_pt_sodreab)+(SGLT2_Na_reabsorption - 0.00235)/number_of_functional_tubules;

SN_PT_Na_outflow = SN_filtered_Na_load-SN_proximal_tubule_reabsorption;

PT_Na_outflow = SN_PT_Na_outflow*number_of_functional_tubules;


e_pt_sodreab_adj = SN_proximal_tubule_reabsorption/SN_filtered_Na_load;




  #SN_proximal_tubule_reabsorption = min(SN_filtered_Na_load,renal_threshold_Na_reabs)*e_pt_sodreab; #SN absolute PT sodium reabsorption

  #SN_PT_Na_outflow = SN_filtered_Na_load*(1-e_pt_sodreab); #SN sodium flow out the proximal tubule into the loop of henle
```

#PT_Na_outflow = SN_PT_Na_outflow*number_of_functional_nephrons; #Total PT sodium flow out of the proximal tubule

#Loop of Henle sodium reabsorption

e_loh_sodreab = nominal_loh_na_reabsorption*(1-0.65)*pressure_natriuresis_LoH_effect; #LoH fractional rate of reabsorption

SN_LoH_reabsorption = SN_PT_Na_outflow*nominal_loh_na_reabsorption*pressure_natriuresis_LoH_effect; #SN absolute LoH sodium reabsorption

SN_LoH_outflow = SN_PT_Na_outflow*(1-nominal_loh_na_reabsorption*pressure_natriuresis_LoH_effect); #SN sodium flow out of the LoH, into the MD

#Macula Densa sodium flow and TGF signal

SN_macula_densa_Na_flow = SN_LoH_outflow;

macula_densa_Na_flow = SN_macula_densa_Na_flow*number_of_functional_tubules;

TGF0_tubulo_glomerular_feedback = 1 - S_tubulo_glomerular_feedback/2;

tubulo_glomerular_feedback_signal = (TGF0_tubulo_glomerular_feedback + S_tubulo_glomerular_feedback / (1 + exp((F0_TGF - SN_macula_densa_Na_flow*baseline_nephrons)*(2e6/baseline_nephrons) / F_md_scale_tubulo_glomerular_feedback)));

#Distal convoluted tubule sodium reabsorption

e_dt_sodium_reabsorption = nominal_dt_na_reabsorption * pressure_natriuresis_DT_effect;

SN_distal_tubule_Na_reabsorption = (SN_macula_densa_Na_flow * min(1,e_dt_sodium_reabsorption));

distal_tubule_Na_reabsorption = SN_distal_tubule_Na_reabsorption*number_of_functional_tubules;

SN_distal_tubule_Na_outflow = (SN_macula_densa_Na_flow - SN_distal_tubule_Na_reabsorption);

distal_tubule_Na_outflow = SN_distal_tubule_Na_outflow*number_of_functional_tubules;

```
  #Collecting Duct sodium reabsorption

  e_cd_sodreab = min(1,nominal_cd_na_reabsorption * pressure_natriuresis_CD_effect);

  SN_collecting_duct_Na_reabsorption = min(CD_Na_reabs_threshold,(SN_distal_tubule_Na_outflow * min(1,e_cd_sodreab)));

  collecting_duct_Na_reabsorption = SN_collecting_duct_Na_reabsorption*number_of_functional_tubules;


  #Total Sodium excretion

  Na_excretion_via_urine = (SN_distal_tubule_Na_outflow - SN_collecting_duct_Na_reabsorption)*number_of_functional_tubules;


  #Sodium Balance

  Na_balance = Na_intake - Na_excretion_via_urine;

  FENA = Na_excretion_via_urine/filtered_Na_load; #Fractional Excretion of sodium


  ###Tubular fluid flow rates


  #Tubular Dimensions (the rest are specified in the parameters file

  L_pt_conv = L_pt_conv_nom*(1+tubular_length_increase);

#L_pt_conv = L_pt_conv_nom;

  L_pt_recta = L_pt_recta_nom*(1+tubular_length_increase);

  #L_pt_recta = L_pt_recta_nom;

  Dc_pt = Dc_pt_nom*(1+tubular_diameter_increase);

#Dc_pt = Dc_pt_nom;

  L_pt = L_pt_conv+L_pt_recta;
```

######## Urine albumin (albumin excretion rate)

signal_glomerular_press= (glomerular_pressure-nom_glomerular_pressure);

fractional_glomerular_press= max(0,((glomerular_pressure-2)/nom_glomerular_pressure)-1);

sieving_coefficient_change= fractional_glomerular_press / T_gp_seiving;

albumin_sieving_coefficient= nom_albumin_sieving_coefficient + sieving_coeff_increase;

SN_albumin_filtation_rate =
nom_plasma_albumin_concentration*SNGFR_nL_min*albumin_sieving_coefficient* mL_nL* dl_ml*1e-6;
#microgm/min

SN_albumin_reabsorption = min(max_SN_albumin_reabsorption, SN_albumin_filtation_rate*
PT_albumin_reabsorption_fraction);   #microgm/min

SN_albumin_excretion_rate = (SN_albumin_filtation_rate -
SN_albumin_reabsorption)*number_of_functional_tubules*24*60*1e3;   #miligm/day

albumin_excretion_rate = (nom_albumin_excretion+SN_albumin_excretion_rate); #mg/24h

###Effect of glomerular pressure on nephron loss

nephron_loss_rate = (1 - fraction_nephrons_lost)* max(0,fractional_glomerular_press) /
T_gp_nephron_loss;

################## Added for tubular growth #########################

```
#Tubular sodium reabsorption per unit SA as the driver of tubular hypertrophy

PT_Na_reab_perUnitSA =
SN_filtered_Na_load*e_pt_sodreab_adj/(3.14*Dc_pt*(L_pt_conv+L_pt_recta));


normalized_PT_reabsorption_density = PT_Na_reab_perUnitSA/PT_Na_reab_perUnitSA_0;

PT_Na_reabs_effect_increasing_tubular_length = (maximal_tubule_length_increase -
tubular_length_increase) * max(normalized_PT_reabsorption_density - 1,0) / T_PT_Na_reabs_PT_length;

PT_Na_reabs_effect_increasing_tubular_diameter = (maximal_tubule_diameter_increase -
tubular_diameter_increase) * max(normalized_PT_reabsorption_density - 1,0) /
T_PT_Na_reabs_PT_diameter;


###################### end ###################


################## Added for change in K_f ##########################


#normalized_ultra_filtration_coefficient = (glomerular_pressure-1.5)/nom_glomerular_pressure;


#ultra_filtration_coefficient_change=(maximal_ultra_filtration_coefficient_change-
ultra_filtration_coefficient_increase)* max(normalized_ultra_filtration_coefficient - 1,0) /
T_ultra_filtration_coefficient;


normalized_kf = (glomerular_pressure-1.05)/nom_glomerular_pressure;


kf_surface_change=(maximal_kf_surface_change-kf_surface_increase)* max(normalized_kf - 1,0) /
T_kf_surface;

kf_permeability_change=(maximum_kf_permeability_change-kf_permeability_increase)*
max(normalized_kf - 1,0) / T_kf_permeability;
```

```
###################### end ###################

gamma =  1.16667e-5; #fluid viscosity

mmHg_Nperm2_conv = 133.32;

pi=3.14;


#Tubular water reabsorption rates

A_pt = -log(1-e_pt_sodreab)/(L_pt_conv+L_pt_recta);

A_lh_des =  -log(1-nominal_loh_na_reabsorption)/L_lh_des;

A_lh_asc = 1;

A_dt = -log(1-e_dt_sodium_reabsorption)/L_dt;


#Tubular control pressures (See Jensen 1964)


Pc_pt_conv = Pc_pt_conv_mmHg*mmHg_Nperm2_conv;

Pc_pt_recta = Pc_pt_recta_mmHg*mmHg_Nperm2_conv;

Pc_lh_des = Pc_lh_des_mmHg*mmHg_Nperm2_conv;

Pc_lh_asc = Pc_lh_asc_mmHg*mmHg_Nperm2_conv;

Pc_dt = Pc_dt_mmHg*mmHg_Nperm2_conv;

Pc_cd = Pc_cd_mmHg*mmHg_Nperm2_conv;

P_interstitial = (nom_postglomerular_pressure)*mmHg_Nperm2_conv;


#Tubular fluid flow rates
```

F_out_pt_conv = (SNGFR_nL_min*1e-12)*exp(-A_pt*L_pt_conv); #Flow out of the proximal convoluted tubule

F_out_pt_recta = F_out_pt_conv*exp(-A_pt*L_pt_recta); #Flow out of the PT recta

F_out_lh_des = F_out_pt_recta*exp(- A_lh_des*L_lh_des); #Flow out of the descending loop of henle

F_out_lh_asc = F_out_lh_des; #Flow out of the ascending loop of henle (impermeable to water)

F_out_dt = F_out_lh_asc*exp(- A_dt*L_dt); #Flow out of the distal tubule

#Flow out of the collecting duct is a function of fluid delivery, the fractional rate of sodium reabsorption, and the vasopressin concentration

F_out_cd = max(F_out_dt*(1-(e_cd_sodreab*normalized_vasopressin_concentration)),0);

#Total flow rates

urine_flow_rate = F_out_cd*1e12*number_of_functional_tubules*1e-6/1000;

daily_urine_flow = (urine_flow_rate * 60 * 24);

Tubule pressure (see Jensen 1964)

#Pressure at the inlet to the distal tubule

B1 = (4*tubular_compliance+1)*128*gamma/pi;

B2_dt = (Pc_dt^(4*tubular_compliance))*F_out_lh_asc/((Dc_dt^4)*A_dt);

P_in_dt = (Pc_cd^(4*tubular_compliance+1)+B1*B2_dt*(1-exp(-A_dt*L_dt)))^(1/(4*tubular_compliance+1));

P_in_dt_mmHg = (P_in_dt+P_interstitial)/mmHg_Nperm2_conv;

#Pressure at the inlet to the ascending loop of henle

B2_lh_asc = (Pc_lh_asc^(4*tubular_compliance))*F_out_lh_des/((Dc_lh^4)*A_lh_asc);

P_in_lh_asc = (P_in_dt^(4*tubular_compliance+1)+B1*B2_lh_asc*(1-exp(-A_lh_asc*L_lh_asc)))^(1/(4*tubular_compliance+1));

P_in_lh_asc_mmHg = (P_in_lh_asc+P_interstitial)/mmHg_Nperm2_conv;

#Pressure at the inlet to the descending loop of henle

B2_lh_des = (Pc_lh_des^(4*tubular_compliance))*F_out_pt_recta/((Dc_lh^4)*A_lh_des);

P_in_lh_des = (P_in_lh_asc^(4*tubular_compliance+1)+B1*B2_lh_des*(1-exp(-A_lh_des*L_lh_des)))^(1/(4*tubular_compliance+1));

P_in_lh_des_mmHg = (P_in_lh_des+P_interstitial)/mmHg_Nperm2_conv;

#Pressure at the inlet to the PT recta

B2_pt_recta = (Pc_pt_recta^(4*tubular_compliance))*F_out_pt_conv/((Dc_pt^4)*A_pt);

P_in_pt_recta= (P_in_lh_des^(4*tubular_compliance+1)+B1*B2_pt_recta*(1-exp(-A_pt*L_pt_recta)))^(1/(4*tubular_compliance+1));

P_in_pt_recta_mmHg = (P_in_pt_recta+P_interstitial)/mmHg_Nperm2_conv;

#Bowman's pressure, which is also the pressure at the inlet to the Proximal convoluted tubule

B2_pt_conv = (Pc_pt_conv^(4*tubular_compliance))*(SNGFR_nL_min*1e-12)/((Dc_pt^4)*A_pt);

P_in_pt_conv= (P_in_pt_recta^(4*tubular_compliance+1)+B1*B2_pt_conv*(1-exp(-A_pt*L_pt_conv)))^(1/(4*tubular_compliance+1));

P_in_pt_conv_mmHg = (P_in_pt_conv+P_interstitial)/mmHg_Nperm2_conv;

###Peritubular oncontic pressure

#Oncotic pressure into the peritubular equals oncotic pressure out of the glomerular capillaries

Oncotic_pressure_peritubular_in = Oncotic_pressure_out;

#Plasma protein concentration existing the kidney depends on RBF and amount of fluid filtered and not reabsorbed as the blood passes through the kidney, which is basically the urine flow rate

plasma_protein_concentration_peritubular_out = (SNRBF_nl_min)*plasma_protein_concentration/(SNRBF_nl_min-urine_flow_rate*1e6*1000/number_of_functional_nephrons);

#Oncotic pressure at the end of the peritubular capillaries is a function of the plasma protein concentration leaving the kidney

Oncotic_pressure_peritubular_out = 1.629*plasma_protein_concentration_peritubular_out+0.2935*(plasma_protein_concentration_peritubular_out^2);

#Average oncotic pressure calculated, assuming oncotic pressure drops linearly along the peritubular capillaries.

oncotic_pressure_peritubular_avg = (Oncotic_pressure_peritubular_in+Oncotic_pressure_peritubular_out)/2;

#Total fluid reabsorbed back into the peritubular capillaries is GFR (in L/min) - urine flow rate (in L/min), since everything that is filtered but not excreted must return to the peritubular capillaries

tubular_reabsorption = GFR_ml_min/1000 - urine_flow_rate;

#According to starlings law, tubular reabsorption = Kf*(Peritubular Capillary Pressure - Oncotic pressure different - RIHP)

#Therefore, RIHP = Peritubular capillary pressure - (peritubular oncotic pressure - interstitial oncotic pressure) + tubular reabsorption/ Kf

RIHP = postglomerular_pressure - (oncotic_pressure_peritubular_avg - interstitial_oncotic_pressure) + tubular_reabsorption/nom_peritubular_cap_Kf;

###Differential equations

#These equations simply provide a sligthly delayed copy of the original variable, so that that variable can be used in equations the appear above where the variable is first defined

d/dt(pre_renal_blood_pressure) = C_prerenal_blood_pressure * (mean_arterial_pressure_MAP - pre_renal_blood_pressure);

d/dt(tubulo_glomerular_feedback_effect) = C_tgf * (tubulo_glomerular_feedback_signal-tubulo_glomerular_feedback_effect);

d/dt(preafferent_pressure_autoreg_signal) = 500*(preafferent_pressure_autoreg_function - preafferent_pressure_autoreg_signal);

d/dt(glomerular_pressure_autoreg_signal) = 500*(glomerular_pressure_autoreg_function - glomerular_pressure_autoreg_signal);

d/dt(F_out_dt_delay) = 100*(F_out_dt - F_out_dt_delay);

d/dt(P_bowmans) = C_P_bowmans*(P_in_pt_conv_mmHg - P_bowmans);

d/dt(oncotic_pressure_difference) = C_P_oncotic*(oncotic_pressure_avg - oncotic_pressure_difference);

d/dt(renal_blood_flow_L_min_delayed)=C_rbf*(renal_blood_flow_L_min - renal_blood_flow_L_min_delayed);

d/dt(renal_interstitial_hydrostatic_pressure) = C_rihp*(RIHP - renal_interstitial_hydrostatic_pressure);

d/dt(tubular_length_increase) = PT_Na_reabs_effect_increasing_tubular_length;

d/dt(tubular_diameter_increase) = PT_Na_reabs_effect_increasing_tubular_diameter;

d/dt(kf_surface_increase) = kf_surface_change;# Change in K_f

d/dt(kf_permeability_increase) = kf_permeability_change;# Change in K_f

d/dt(sieving_coeff_increase) = sieving_coefficient_change;

d/dt(fraction_nephrons_lost) = nephron_loss_rate;

d/dt(preafferent_resistance_decrease) = preafferent_change;

#Extracellular fluid volume = water in - urine out

d/dt(extracellular_fluid_volume) = C_water_intake_ecf_volume * (water_intake) + C_urine_flow_ecf_volume * (urine_flow_rate);

#Total body sodium = sodium intake - sodium excretion

d/dt(sodium_amount) = C_na_excretion_na_amount * (Na_excretion_via_urine) + C_na_intake_na_amount * (Na_intake);

#These signals serve as input to the whole body autoregulation controller. The choice of C in these equation will determine the speed of this autoregulatory response

d/dt(cardiac_output_delayed) = C_cardiac_output_delayed*(cardiac_output - cardiac_output_delayed);

d/dt(CO_error) = C_co_error*(cardiac_output-CO_nom);

#This signal serves as input to vasopressin controller. The choice of C will determine the speed of this response

d/dt(Na_concentration_error) = C_Na_error*(Na_concentration - ref_Na_concentration);

#F0_TGF is the setpoint for the TGF response. When C_tgf_reset = 0, TGF will not reset. When C_tgf_reset is >0, the TGF setpoint will reset to the ambient MD sodium flow level

#The value of C_tgf_reset determines the speed of this response.

d/dt(F0_TGF) = C_tgf_reset*(SN_macula_densa_Na_flow*baseline_nephrons - F0_TGF);

d/dt(serum_creatinine) = creatinine_synthesis_rate - creatinine_clearance_rate;

d/dt(glucose_concentration) = glucose_concentration_change;

d/dt(Na_intake) = Na_intake_rate_change;

"

```
save(ode, file = "model_struct.saved")
```

Model Parameters

```
calcNomParams <- function(){

#Constants and Unit conversions
nL_mL=1e+06
mL_nL = 1e-6
dl_ml=0.01
L_dL=10
L_mL=1000
L_m3=0.001
g_mg=0.001
ng_mg=1e-06
secs_mins=60
min_hr=60
hr_day=24
min_day=1440
MW_creatinine=113.12
Pi=3.1416
viscosity_length_constant=1.5e-09
albumin_excr_parameter=4.1e5
```

```
#######################################################################################
##

#Parameters of normal human physiology based on literature and commmon medical knowledge

#######################################################################################
##


#Scaling parameters - can be used to parameterize model for other species

ECF_scale_species = 0.0004

BV_scale_species=1e-6 #needs to change

water_intake_species_scale = 1#.0025

CO_scale_species = 8e-4

#maximal_tubule_length_increase=1


####Systemic parameters

nominal_map_setpoint=101        #mmHg #Changed for Mice

CO_nom= 0.004                   #L/min #Changed for Mice

ECF_nom = 0.0058                #L #Changed for Mice

Na_intake_rate=0.0004           #mEq/min  #Changed for Mice

nom_water_intake = 0.005        #L/day  #adjusted for species using water_intake_species_scale

ref_Na_concentration=140        #mEq/L

plasma_protein_concentration = 4#3.42 #g/dl #Changed for Mice

plasma_albumin_concentration= 723      #g/dl


nominal_glucose_concentration= 100 #mg/dl

maximum_glucose_concentration = nominal_glucose_concentration

T_glucose = 0.1
```

```
glucose_mg_mmol=0.00554994394556615#mmol/dl

nom_renal_threshold_glucose_reabsorption=150;

SGLT2_inhibition=1;


equilibrium_serum_creatinine=0.8         #mg/dl

potassium_concentration=5       #mEq/L

P_venous=4       #mmHg

R_venous=3500#mmHg

nom_right_atrial_pressure=0.87 #mmHg

reference_BV_mean_filling_pressure=30.18 #mmHg


####Renal parameters

nom_renal_blood_flow_L_min =0.0018 #0.002#0.0025 #0.0019 #0.00165            #L/min #Changed for Mice

baseline_nephrons=10000 #11000 #Changed for Mice

nom_Kf=3.9  #3.9                                                #nl/(min*mmHg)

nom_oncotic_pressure_difference =11.9            #mmHg

P_renal_vein=4                                  #mmHg

no_of_kidney = 1


nom_oncotic_pressure_peritubular= 12 #mmHg #Changed for Mice

interstitial_oncotic_pressure = 5            #mmHg


#Renal Vasculature

nom_preafferent_arteriole_resistance= 30000 ##15       #mmHg

nom_afferent_diameter=1.3e-5  ###1.5e-05       #mmHg
```

```
nom_efferent_diameter=0.985e-5          #mmHg


#Renal Tubules

Dc_pt_nom  = 27e-6                      #m

Dc_lh = 17e-6                           #m

Dc_dt = 17e-6                           #m

L_pt_conv_nom = 0.0011                  #m

L_pt_recta_nom =0.0011                          #m

L_lh_des = 0.0011                               #m

L_lh_asc = 0.0011                               #m

L_dt = 0.0023                           #m

tubular_compliance = 0.2

Pc_pt_conv_mmHg = 16.8 #15                      #mmHg #Changed for Mice

Pc_pt_recta_mmHg = 12                           #mmHg

Pc_lh_des_mmHg = 11             #mmHg #Changed for Mice

Pc_lh_asc_mmHg = 10                     #mmHg #Changed for Mice

Pc_dt_mmHg = 7.5                                #mmHg

Pc_cd_mmHg = 7.8        #mmHg

nominal_pt_na_reabsorption=0.782        #fraction

nominal_loh_na_reabsorption = 0.4       #fraction

nominal_dt_na_reabsorption=0.5          #fraction



maximal_tubule_length_increase = 0.5

T_PT_Na_reabs_PT_length = 500000
```

```
maximal_tubule_diameter_increase = 0.5

T_PT_Na_reabs_PT_diameter= 500000

maximal_ultra_filtration_coefficient_change=0.5

T_ultra_filtration_coefficient=5000

#PT_Na_reab_perUnitSA_0= 0.11172e6

maximal_kf_surface_change=0.5

maximum_kf_permeability_change=1

T_kf_surface=750

T_kf_permeability= 40000


max_preafferent_decrease = 0.25

T_preafferent = 100




nom_plasma_albumin_concentration = 3.5        #g/dl

nom_albumin_sieving_coefficient = 0.0006

nom_albumin_excretion = 0.075# mg/day

PT_albumin_reabsorption_fraction = 0.995

max_SN_albumin_reabsorption = 2.5e-6

albumin_diabetic_parameter = 1.0

no_of_kidney = 1.0 # this will be 0.5 for uninephroctomized mice.

GP_albumin_slope = 4e4


##########################################################################################
##
```

#The following parameters are calculated at equilibrium using the parameters above

##
##

#This pressure is the setpoint that determines the myogenic response of the preafferent vasculature

nom_preafferent_pressure = nominal_map_setpoint - nom_renal_blood_flow_L_min*nom_preafferent_arteriole_resistance;

#This pressure is the setpoint that determines the myogenic response of the afferent vasculature

nom_glomerular_pressure = nom_preafferent_pressure - nom_renal_blood_flow_L_min*(L_m3*viscosity_length_constant/(nom_afferent_diameter^4)/baseline_nephrons);

#This pressure is the setpoint that determines the tubular pressure-natriuresis response

nom_postglomerular_pressure = nom_preafferent_pressure - nom_renal_blood_flow_L_min*(L_m3*viscosity_length_constant*(1/(nom_afferent_diameter^4)+1/(nom_efferent_diameter^4))/baseline_nephrons);

The rate of sodium excretion must equal the rate of sodium intake. Sodium reabsorption rates vary along the tubule, but based on literature

measurements we have a good, and literature data provides estimates for these rates. However, there is a precise

rate of sodium reabsorption required to achieve the equilibrium defined by the parameters above.

Assuming that reabsorption rates are known in all but one segment of the tubule, the exact rate

# of reabsorption of the remaining segment can be calculated. We chose to calculate the CD rate of reabsorpion based on estimates for

PT, LoH, and DT reabsorption.

nom_GFR = nom_Kf*(nom_glomerular_pressure - nom_oncotic_pressure_difference - Pc_pt_conv_mmHg)/nL_mL*baseline_nephrons;

nom_filtered_sodium_load = nom_GFR/L_mL*ref_Na_concentration;

nom_PT_Na_outflow = nom_filtered_sodium_load*(1-nominal_pt_na_reabsorption);

nom_LoH_Na_outflow = nom_PT_Na_outflow*(1-nominal_loh_na_reabsorption);

nom_DT_Na_outflow = nom_LoH_Na_outflow*(1-nominal_dt_na_reabsorption);

nominal_cd_na_reabsorption = 1-Na_intake_rate/nom_DT_Na_outflow;

#RBF = (MAP - P_venous)/RVR. Given MAP, P_venous, RBF, and preafferent, afferent, and efferent resistances, the remaining peritubular resistance at steady state can be determined

nom_RVR = (nominal_map_setpoint - P_venous)/nom_renal_blood_flow_L_min

nom_peritubular_resistance = nom_RVR - (nom_preafferent_arteriole_resistance + L_m3*viscosity_length_constant*(1/nom_afferent_diameter^4+1/nom_efferent_diameter^4)/baseline_nephrons);

#Calculate the normal amount of sodium reabsorbed per unit surface area of the PT

PT_Na_reab_perUnitSA_0 = (nom_filtered_sodium_load/baseline_nephrons)* nominal_pt_na_reabsorption/(3.14*Dc_pt_nom*(L_pt_conv_nom+L_pt_recta_nom))

#Given the values for baseline MAP and CO above, the baseline TPR required to maintain this MAP and CO can be calculated. Since TPR includes renal vascular resistance, the baseline systemic (non-renal) resistance

```
#can be calculated from this TPR and the values for baseline renal resistances defined above.

nom_TPR = nominal_map_setpoint/CO_nom

#nom_systemic_arterial_resistance= (nom_TPR-R_venous)*nom_RVR/(nom_RVR+R_venous - nom_TPR)

nom_systemic_arterial_resistance = nom_TPR-R_venous


#

tubular_reabsorption = nom_GFR/1000 - nom_water_intake*water_intake_species_scale/60/24  #at SS, water excretion equals water intake

RIHP0 = nom_postglomerular_pressure  #Both RIHP and Kf are unknown, so we can either assume RIHP and calculate Kf, or vice versa. Since RIHP has been measured experimentally,

                        #it seems better to assume a normal value for RIHP and calculate Kf

RIHP0 = nom_postglomerular_pressure

nom_peritubular_cap_Kf = - tubular_reabsorption/(nom_postglomerular_pressure - RIHP0 - (nom_oncotic_pressure_peritubular - interstitial_oncotic_pressure))


###############################################################################################
##

#The following parameters were determined indirectly from many different literature studies on the response

#various changes in the system (e.g. drug treatments, infusions of peptide, fluid, sodium, etc.....)

###############################################################################################
##


#Effects of Renal Sympathetic Nerve Activity on preafferent resistance, renin secretion, and PT sodium reabsorption

#nom_rsna = 1

#map_rsna_slope=5 #
```

```
#rap_rsna_slope=0.008

#rsna_aff_slope = 0.7

#rsna_aff_scale = 5


#Effects of AT1-bound AngII on preafferent, afferent, and efferent resistance, and aldosterone secretion

AT1_svr_slope = 1e-10

AT1_preaff_slope =  1e-10

AT1_aff_slope= 0.005#1e-10#0.005

AT1_eff_slope = 0.01# 1e-10#0.01

AT1_PT_slope =  0.002#1e-10#0.002 #0.00065

AT1_aldo_slope =  0.02#1e-10#0.02


#Effects of Aldosterone on distal and collecting duct sodium reabsorption

nominal_aldosterone_concentration=85

hill_aldo_DT=0.1

scale_aldo_DT=0#1

hill_aldo_CD=0.02

scale_aldo_CD=0#1


#Effects of Atrial Natriuretic Peptide (ANP)preafferent, afferent, and efferent resistance and collectin
duct sodium reabsorption

nom_ANP=1

rap_anp_slope= 1e-10#1

anp_aff_resistance_slope=0.4125# 1e-10#0.4125

anp_eff_resistance_slope=0.0825# 1e-10#0.0825

anp_preaff_resistance_slope=0.4125# 1e-10#0.4125
```

anp_cd_slope = 0.055#1e-10#0.055

#Effects of Renal Sympathetic Nerve Activity on preafferent resistance, renin secretion, and PT sodium reabsorption

nom_rsna = 1

map_rsna_scale=1#1

map_rsna_slope= 5#1e-10#5

rap_rsna_slope= 0.008#1e-10#0.008

rsna_aff_slope = 0.7#1e-10#0.7

rsna_aff_scale = 1#5

rsna_preaff_vmax=0 #1.5,

rsna_preaff_EC50 = 1

rsna_preaff_hill = 4

#rsna_renin_slope=0.36

pt_rsna_scale=1

pt_rsna_slope= 1e-10

#Osmolarity control of vasopressin secretion

Na_controller_gain=2

Kp_VP = 0.1

Ki_VP = 0.01

#Effects of Vasopressin on water intake and reabsorption

nominal_vasopressin_conc=4

water_intake_vasopressin_scale = 1.5

water_intake_vasopressin_slope = -0.5

#Magnitude and Steepness of tubuloglomerular feedback

S_tubulo_glomerular_feedback=0.6

F_md_scale_tubulo_glomerular_feedback=0.5

#Responsiveness of renal vasculature to regulatory signals

preaff_diameter_range=0.25

afferent_diameter_range=1.2e-05

efferent_diameter_range=3e-06

preaff_signal_nonlin_scale=3

afferent_signal_nonlin_scale=3

efferent_signal_nonlin_scale=3

#Limit on PT sodium reabsorption

renal_threshold_Na_reabs = 16e-6

#Empirical relationship between blood volume and cardiac filling pressure - from Guyton

BV_filling_pressure_slope=7.436

#Transfer constants for ODEs - determine speed of processes

C_prerenal_blood_pressure=1000

C_P_bowmans = 1000

C_P_oncotic = 1000

C_rbf=1000

C_tgf_reset=0

C_cardiac_output_delayed=.001

C_co_error=0.00001

C_rihp = 0.1#0.01 #time delay between peritubular pressure and RIHP

C_tgf=1/30 #1000

C_na_excretion_na_amount=-1/30

C_na_intake_na_amount=1/30

C_urine_flow_ecf_volume=-1/30

C_water_intake_ecf_volume=1/30

C_Na_error=1/60

##

#These parameters are by default set to ensure strong autoregulation of cardiac output, RBF, glomerular pressure, and MAP

#However, reducing these parameters reduces the ability of the system to autoregulate, and is necessary for modeling the development of hypertension, etc.

##

```
#Metabololic tissue autoregulation of cardiac output

tissue_autoreg_scale=1

Kp_CO=1.5

Ki_CO=30



#Renal autoregulation of glomerular pressure

gp_autoreg_scale = 1e-10

preaff_autoreg_scale = 0.1

myogenic_steepness = 2


#Renal autoregulation of renal blood flow

RBF_autoreg_scale = 0#3

RBF_autoreg_steepness=0.001


#Pressure natiuresis effect through collecting duct sodium reabsorption

#Parameters selected based on Isaksson 2014:

#For a 10X increase in salt intake:MAP increases by 5mmHg, Renin decreases by 45%

#GFR increases by 1.4ml/min

#Strong CD effect required to minimize BP rise

#PT effect + LoH effect required to produce renin response

#If PT effect is too big, GFR will decrease instead of increase.

#So LoH must make up for the rest

pressure_natriuresis_CD_scale = 0.25#33#2

pressure_natriuresis_CD_slope=0.1
```

```
pressure_natriuresis_PT_scale =0#.2
pressure_natriuresis_PT_slope = 0.1

pressure_natriuresis_LoH_scale = 0.1#1
pressure_natriuresis_LoH_slope = 0.1

pressure_natriuresis_DT_scale = 0.1#1
pressure_natriuresis_DT_slope = 0.1


creatinine_synthesis_rate  = equilibrium_serum_creatinine * dl_ml * nom_GFR #Units: mg/min


t=sort(ls())
param=sapply(t,names)
for (i in 1:length(t)){
  param[i]=get(t[i])
}
param$param=NULL

return(param)
}
```
